# Why do Middle-Aged Adults Report Worse Mental Health and Wellbeing than Younger Adults? An Exploratory Network Analysis of the Swiss Household Panel Data

**DOI:** 10.1007/s11482-024-10274-4

**Published:** 2024-04-04

**Authors:** Dawid Gondek, Laura Bernardi, Eoin McElroy, Chiara L. Comolli

**Affiliations:** 1 Swiss Centre of Expertise in Life Course Research (LIVES), University of Lausanne, Bâtiment Géopolis, CH-1015 Lausanne, Switzerland; 2 FORS, Swiss Centre of Expertise in the Social Sciences, Lausanne, Switzerland; 3 School of Psychology, Ulster University, Coleraine, UK; 4 Department of Statistics “Paolo Fortunati”, University of Bologna, Bologna, Italy

**Keywords:** Mental Health, Wellbeing, Midlife, Network Analysis, Life Course

## Abstract

Despite the growing consensus that midlife appears to be a particularly vulnerable life phase for lower mental health and wellbeing, little is known about the potential reasons for this phenomenon or who the individuals at higher risk are. Our study used six waves (2013–2018) of the Swiss Household Panel (n = 5,315), to compare the distribution of mental health and wellbeing, as well as their key correlates, between midlife (40–55 years) and younger adults (25–39 years) in Switzerland. Moreover, using network analysis to investigate interrelationships across life domains, we describe the complex interrelations between multiple domain-specific correlates and indicators of both mental health and wellbeing across the two age groups. Middle-aged (age 40–55) individuals reported lower life satisfaction and joy, as well as higher anger, sadness, and worry than young adults (age 25–39), with the effect sizes reaching up to 0.20 Cohen’s d. They also reported lower social support, relationships satisfaction, health satisfaction, and higher job demands and job insecurity. Relationships satisfaction and social support were the most consistent correlates across all three indicators of wellbeing in both age groups. Health satisfaction was more strongly, and directly, interrelated with energy and optimism in midlife compared with young adulthood (0.21 vs 0.12, p = 0.007). Job demands were more strongly linked with anger and sadness in midlife. The network model helped us to identify correlates or their clusters with direct and strong links to mental health and wellbeing. We hypothesised that health satisfaction, relationships satisfaction, social support, and job demands may help to explain worse mental health and wellbeing in midlife.

**Supplementary Information:**

The online version contains supplementary material available at 10.1007/s11482-024-10274-4.

## Introduction

At the population level, mental health and wellbeing tend to deteriorate between early-30s and midlife (40–55 years of age) – this is the most consistent finding in the context of life course trajectories of these health indicators. This age trend has been shown by longitudinal studies in multiple Western countries, including Great Britain, France, Switzerland and the United States of America (USA), and across various birth cohorts (Barbuscia & Comolli, 2021; Blanchflower, 2021; Giuntella et al., 2022; Gondek et al., 2021a, 2021b). Moreover, middle-aged individuals (mainly men) are at the greatest risk of dying from suicide and they do not respond to psychotherapy as well as other age groups (Case & Deaton, 2015; Cuijpers et al., 2020; Office for National Statistics, 2019).

Interestingly, most of the debate around life course trajectories of mental health and wellbeing is concentrated on the change between midlife and older age (Blanchflower & Graham, 2021, 2022; Galambos et al., 2020). For instance, some argued that an apparent improvement in mental health between these life stages is a statistical artefact, due to age changes being confounded by cohort effects or survival bias (Galambos et al., 2020). The scepticism remains that wellbeing can increase in older age, while being accompanied by poor health, loneliness, death of family members and friends. While wellbeing and mental health at older age received substantial attention, there is hardly any research on the change in these indicators between young and middle adulthood. This is despite these outcomes going from the highest to the lowest within around 10 years. Our study helps to uncover complex life course processes that may contribute to worse mental health and wellbeing in midlife, compared to earlier age.

Specifically, we have three objectives. First, using the Swiss Household Panel (SHP), we compare the distribution of mental health and wellbeing between midlife (40–55 years) and younger adulthood (25–39 years). Secondly, we compare the distribution of identified correlates of mental health and wellbeing between young and midlife adults. This will help us to understand the degree to which different life domains reflect risk and protective factors for mental health, and whether this varies between young adulthood and midlife. Thirdly, we visually describe the complex interrelations between multiple correlates, spanning various life domains, and indicators of both mental health and wellbeing simultaneously. To meet this objective, we use network analysis, which has been gaining popularity in health and social sciences. Network analysis is a data-driven exploratory method, which can inform predictive (who is at heightened risk of poor mental health in midlife?) and explanatory (why midlife is associated with an elevated risk of poor mental health?) research (Schmueli, 2010).

### Theoretical Framework for Identifying Correlates of Mental Health and Wellbeing

The aim of the theoretical framework in this study is to identify the key variables available in the SHP, which are potentially important for mental health and wellbeing. As this is an exploratory study, we do not draw any specific hypotheses in terms of their relationship with mental health and wellbeing (Scheel et al., 2020; Schmueli, 2010). Instead, we estimate and visually describe the relative importance of these correlates in midlife, when mental health and wellbeing are expected to be poorer, compared to young adulthood. We wish to generate data-driven hypotheses helping to explain the increased risk of poor mental health and wellbeing in midlife compared with earlier age. These hypotheses could be tested by confirmatory research, for instance, using causal analysis of observational studies.

This objective is motivated by the lack of comprehensive theories guiding research on poor mental health and wellbeing in midlife. The most prominent theory was proposed by Jaques (1965), who coined the psychological concept of a ‘midlife crisis’. Jacques proposed that midlife crisis is evoked by the realisation of upcoming certain death. This can be accompanied by awareness of one’s limitations, restricted possibilities, or unmet ambitions. However, this theory does not explain why most individuals do not suffer from a dip in mental health or wellbeing in midlife (Gondek et al., 2022). Hence it has a limited practical utility in distinguishing which individuals are at a lower and higher future risk, or why, on average, lower levels of mental health and wellbeing are observed at the population level. Also, this theory does not propose any actionable factors that could be targeted by public policies or interventions. There is plentiful of research that investigates the relationship between various life events, such as employment, divorce, childbirth, children moving out of the house and mental health or wellbeing (Tibubos et al., 2021). However, these are not limited to occurring at any given life stage and are heavily affected by cohort effects, where the age trend of drop in mental health between early and middle adulthood remains universal across time (Gondek et al., 2021a, 2021b). For instance, employment becomes increasingly unstable throughout the entire life course, people tend to have children and move out from the parental home at older and older age (Barbieri, 2009; News, 2016; swissinfo.ch, 2020). It is more likely that a drop in wellbeing in midlife is due to a unique combination or accumulation of these factors, about which the current literature tells us very little. Typically, studies examining the links between these events and later mental health or wellbeing include all ages, with age being treated as a confounding factor, or with studies being limited to narrow age period (Thomas et al., 2019; Tibubos et al., 2021).

Despite network analysis being a data-driven approach, it is necessary to decide what variables are to be included in the analysis, which can be based on theory or expert knowledge. Hence, to identify potentially relevant correlates of mental health and wellbeing, we opted for using the framework proposed by the World Health Organisation, which outlines three broad types of determinants of mental health and wellbeing: 1) individual factors, 2) social and economic circumstances, and 3) structural factors (World Health Organization, 2012). Individual factors (including attributes and behaviours) comprise, for instance, self-esteem, communication skills, and health behaviours. Social and economic circumstances determine the extent to which the immediate social surroundings are bolstering or hindering an individual’s capacity to develop and flourish. These comprise economic security, social support, or work stress. Finally, structural factors define the wider socio-cultural and geopolitical environment in which people live, including access to basic services, discrimination, equality or social justice. We choose the WHO framework due to its relative simplicity, wide-recognition, and ease of adaptation to a wide range of life domains. Moreover, this framework emphasises that these determinants interact with each other in a dynamic way (World Health Organization, 2012), which justifies the use of the network analysis in our study. Finally, having to select several correlates to investigate, we decided to focus on those that are, at least some extent, malleable by potential policies and interventions – which is the focus of the WHO framework.

### Why are we Interested in Midlife?

In our study, we specify midlife as being between 40 and 60 years old, with most definitions falling into this age bracket (Lachman, 2015). In line with this definition, adults between 24 and 75 years old from the Midlife in the United States (MIDUS) national longitudinal study reported on average that midlife begins at 44 and ends at 59 years of age (Lachman, 2015). However, it is important to acknowledge that there is no consensus on how midlife should be defined. The operationalisation using chronological age is convenient, but definitions around roles (e.g., a parent) or timing of life events may be more suitable in some contexts due to increasing life and health expectancy, staying longer in education, delaying parenthood and other related changes over time (Lachman, 2015).

Despite the growing evidence on lower levels of population mental health and wellbeing in midlife, particularly compared with younger adulthood (age 25–40), this life phase is often overlooked both in terms of research and policy (Lachman, 2015). For instance, one can easily find multiple recent journal publications, research groups, consortium-written reports, workshops or seminars, and helplines related to mental health of children, young people and elderly (Barrense-Dias et al., 2021; Chastonay et al., 2018; UNICEF, 2021; World Health Organization 2017). However, a similar search leads to very modest results in the context of midlife. This is startling, as midlife is not only a particularly vulnerable life period for poor mental health and wellbeing but it is also often described as a pivotal life phase, characterised by the intersection of growth and decline, the linkage between earlier and later periods of life, and a bridge between younger and older generations (Lachman et al., 2015). Middle-aged individuals can be considered as key decision-makers in society, with great influence on people around them, due to their peak time in career and earnings, contributions to the community and caring responsibilities, including both children and elderly parents (Infurna et al., 2020). But middle-age is also associated with elevated stress, and physical signs of ageing which makes juggling multiple responsibilities difficult (Infurna et al., 2020). This is important from the public mental health perspective as wellbeing of other people, including children, the elderly or co-workers is interdependent with that of midlife individuals. Therefore, a better understanding of and a greater investment in mental health or wellbeing in midlife has the potential to produce cross-over effects on wellbeing of those reliant on the support from the persons in their midlife. This further emphasises that the simultaneous interrelations between multiple life domains, including family, work and individual attributes, should be considered to understand how life changes in these domains across the life course, potentially differentially affect mental health.

Midlife is also a time of opportunities. From the life course perspective, it can be considered as a sensitive period for healthy ageing, when foundations for good mental health in older age can be laid down (Infurna et al., 2020). For instance, recent studies showed that cognitive reserve and social capital accumulated in early and particularly midlife may diminish the harmful influences of mental ill-health on cognitive functioning in old age (Ihle et al., 2018). Taking it all into account, midlife can be characterised as a particularly hectic life stage when both potentially protective and harmful factors for mental health cumulate. Hence, there is a need for an analytical approach able to capture these complex interrelations between multiple protective and harmful factors spanning various life domains. Network analysis provides the necessary flexibility and explorative capacities for such a purpose. The same correlates of mental health and wellbeing, related to individual factors, social circumstances, and the environment in which persons live can manifest themselves at all life stages. However, some of them may be more prevalent or have differing interrelations with mental health or wellbeing in midlife than in other life phases. For instance, physical health problems, which are strongly linked with mental health, tend to increase in midlife, compared to younger age (World Health Organization, 2012). Unemployment, in turn, may be lower in midlife than in young adulthood, but middle-aged individuals can be more disadvantaged in their prospects of re-employment. In addition, the consequences of unemployment may be more severe due to greater financial responsibilities, for instance caring for a family, which could be linked to ill-mental health (OECD, 2022; World Health Organization, 2012).

Prevalence of certain risk factors for midlife wellbeing may, on average, be lower and of protective ones higher in Switzerland than in other Western countries. For instance, Switzerland performs better on most labour market indicators than the Organisation for Economic Co-operation and Development (OECD) countries on average. This includes employment rate, earnings quality, labour market insecurity, job strain, employment gap for disadvantaged groups (OECD, 2018a). Switzerland also performs well in many dimensions of wellbeing relative to other countries, such as education, health, environmental quality, social connections, safety and life satisfaction, disposable income (OECD, 2015). Of particular importance to midlife, in Switzerland, the proportion of employees working very long hours in paid work is much lower than the OECD average (0.4% vs 10%). Despite Switzerland having a favourable socioeconomic context, it appears that like in other countries, young people still tend to be disadvantaged in their job prospects. For instance, young adults (25–39 years) are affected by unemployment to a greater extent than other age groups (25–39 years: 4.4% vs 40–54 years: 3.7%) (Office, 2023). Hence, still differences in wellbeing, for instance, due to job factors could be expected. Moreover, the life transitions, typical for young or middle adulthood, are happening in Switzerland at similar age compared to other Western countries. For instance, Swiss young adults leave parental house one year earlier than the European Union average (25 vs 26) or have children two years later than the OECD average (31 vs 29) (Eurostat, 2021; News, 2016; OECD, 2018b). While these differences are not negligible in other contexts, they should not substantially affect comparisons between broad developmental stages, which were the focus our study.

We explored potential age differences between young and midlife adults in interrelations between correlates and indicators of mental health and wellbeing. We made the comparisons to the age group preceding midlife (25–39 years), as there tends to be a drop in mental health and wellbeing between these ages (Barbuscia & Comolli, 2021; Blanchflower, 2021; Gondek et al., 2021a, 2021b). Hence, potential differences can help us understand the processes leading to this decline in mental health in midlife.

### Rationale for Using a Network Analysis

Network analysis is a method of processing data that allows to visually describe complex structures in a compact and synthetic manner (Epskamp et al., 2018). It can contribute to the main objectives of any health science – prediction and explanation (Schmueli, 2010; Schooling & Jones, 2018).

The purpose of prediction would be to identify correlates that can help to forecast which individuals are at increased risk to suffer from poor mental health and wellbeing in midlife. We could then compare whether these characteristics are similar in young and middle adulthood. This may help to elucidate whether any specific factors may be more predictive in midlife than in younger adulthood. The advantage of network analysis, compared to more traditional models such as stepwise regression, is that network approaches incorporate regularisation techniques that reduce weak correlations towards zero encouraging simpler and sparse models (Hastie et al., 2015).

The aim of explanation in this context is understanding why mental health and wellbeing being are worse in midlife than in younger adulthood. Network analysis, combined with expert knowledge, can help to generate hypotheses about such causes, while offering clues about potential causal dynamics (e.g., presence of additional mediating mechanisms) (Borsboom et al., 2021). These, subsequently, can be tested in causal mediation analysis in a confirmatory manner (Rijnhart et al., 2021). For instance, in a more classic fashion, we can identify specific factors that are strongly and directly linked with mental health or wellbeing. These could be good candidates for potential causes of lower mental health or wellbeing in midlife. Alternatively, there may be clusters (e.g., various indicators of socioeconomic circumstances) or chains of variables (e.g., due to spill-over effects across different life domains) that are linked with mental health and wellbeing.

Applying network analysis allows advancing the efforts in conceptualising life course processes as a complex system, within which aetiological factors mutually reinforce one another, each having either direct or indirect association with mental health and wellbeing (Bernardi et al., 2019; Fried & Cramer, 2017; Galea et al., 2010). As shown by a systematic review of reviews, most of the observational research on determinants of mental health focuses on individual adverse factors or their domains, for instance, socioeconomic circumstances (Lund et al., 2018). Network approach also allows for including multiple indicators of mental health and wellbeing simultaneously, instead of limiting the analysis to a single composite variable. The advantage of such as an approach is that it provides a more holistic picture of relevant interrelations, which could be masked otherwise. This is due to many possibilities of arriving at the same, aggregated, score with high values on some aspects of wellbeing and low on others. Moreover, the associations with a composite may be driven by individual components of the scale (Nielsen et al., 2023). The value of including both wellbeing and mental health measures simultaneously is that they are distinct but core aspects of a person’s functioning (World Health Organization, 1946), with potentially different correlates (Lereya et al., 2022).

In the context of mental health, network analysis has been mainly used in studying the structure of psychopathology, aiming to understand the relationship between various symptoms within and between common mental disorders (Stochl et al., 2019). In terms of research on correlates of mental health, there are two particularly relevant studies, both using a large sample of adults from a socioeconomically disadvantaged region of the United Kingdom (McElroy et al., 2019, 2021). These studies found strong associations between financial difficulties, subjective physical health and wellbeing. Moreover, after controlling for all other variables in the network model, including determinants of socioeconomic circumstances, wellbeing was positively associated with local greenspace usage, civic agency, and neighbourhood cohesion, and negatively associated with housing disrepair (McElroy et al., 2021). The analysis also revealed that drunken/rowdy behaviour was particularly influential within the neighbourhood cluster, and the clusters of mental health symptoms and neighbourhood characteristics were linked primarily by paranoia. These findings emphasised that individual and neighbourhood determinants of wellbeing should not be considered in isolation, as they are interrelated. The network analysis helped to identify potential targets for intervention to improve neighbourhood cohesion (drunken/rowdy behaviour) and mechanisms linking mental health with neighbourhood cohesion (paranoia). However, further research explicitly assessing whether these relationships are causal is needed, particularly as the data were cross-sectional.

To sum up, firstly, we compare the distribution of mental health and wellbeing between midlife (40–55 years) and younger adults (25–39 years) in Switzerland. Secondly, we compare the distribution of key correlates of mental health and wellbeing between young and midlife adults. Thirdly, as the core objective of our study, we visually describe the complex interrelations between multiple correlates, spanning various life domains, and indicators of both mental health and wellbeing simultaneously across the two age groups.

## Methods

The analysis code using in all analyses can be found at https://osf.io/4x36t/?view_only=25d0ddc6de064a14877f8e11f094472e.

### Data

This paper draws on longitudinal data from six waves (2013–2018) of the Swiss Household Panel (SHP) study (Voorpostel et al., 2021). The SHP is a nationally representative household-based panel study that collects yearly information on different aspects of life from each person living in the household at the time of the interview (Voorpostel et al., 2021). Our sample included participants aged 25–55 between 2013 and 2018, who answered the questions on all indicators of mental health and wellbeing and reported being employed at least once in the study period (2013–2018), due to the inclusion of numerous job-related variables. This resulted in a total sample of 5,315 individuals, including 2,044 young (25–39 years) and 3,271 middle-aged (40–55 years) participants.

The survey years of 2013–2018 were chosen for two reasons. First, in 2013 a third replacement sample of the SHP was recruited, hence boosting the sample size. Second, the SHP has a modular design with some questions asked annually and others every three years, hence selecting six waves allowed for the inclusion of at least one measure of each variable of interest. For instance, the life satisfaction questions were asked in 2014 and 2017, whereas satisfaction with living arrangements and relationships satisfaction were measured in 2015 and 2018. If a given variable was reported more than once, we averaged the responses.

### Variables

In the current study, we included six indicators of mental health and wellbeing (anger; sadness; worry; life satisfaction; energy and optimism; joy), along with 24 correlates. According to the framework proposed by the World Health Organisation (2012), the correlates were mapped onto three broad types of determinants of mental health, including individual factors (four correlates, including attributes and behaviours), social circumstances (11 correlates, socioeconomic characteristics and perceived social circumstances), and structural factors (nine correlates, self-perceived factors aggregated at the cantonal level and objectively measured factors). When a given variable was measured more than once, we averaged and rounded the score. As the household income variable was highly positively skewed, it was log-transformed. More details about all variables can be found in Table 1. As one of the assumptions of the network analysis used in our study is a normal distribution of included variables, we excluded several categorical or (zero-inflated) count correlates that are potentially relevant and available in the SHP, such as marital status, home ownership at the individual level, or participation in volunteering activities.

**Table 1:** Description of study variables

Domain	Correlate	Definition
*Individual factors (attributes and behaviours)*	Physical activity	The number of days per week a participant exercises. The answers from between 2013 and 2018 (at least one answer was required) were averaged and rounded to the closest integer
		Participants were asked: “At present, how many days a week do you practise for half an hour minimum a physical activity which makes you slightly breathless?”
	Health satisfaction	Participants were asked in 2014: “How satisfied are you with your state of health, if 0 means "not at all satisfied" and 10 "completely satisfied"?”
		If this information was missing in 2014, it was replaced with reports from 2013 or 2015
	Sense of control	Sense of control was measured with six items in 2015 and 2018—four items on personal mastery (e.g., “Doing everything set in my mind “) and two items on perceived constraints (e.g., “Others determine what I can do”). These items come from existing scales (Lachman & Weaver, 1998; Pearlin & Schooler, 1978). The response options ranged from “completely disagree” (0) to “completely agree” (10)
		The items were averaged separately in 2015 and 2018, with a higher score indicating a greater sense of control. Subsequently, the overall scores from 2015 and 2018 (at least one answer was required) were averaged and rounded to the closest integer
	Self-mastery	Self-mastery refers to how far respondents believe that their destiny is controlled by themselves and their own decisions or by external forces over which they do not have any power. The items capturing self-mastery (e.g., “little influence on life events”) are rated on an eleven-point scale from 0 “I completely disagree” to 10 “I completely agree”. The first four questions are adapted by Levy, Joye, Guye and Kaufmann (p. 510; 1997) (Levy et al., 1997) from Strodtbeck (1958). These items are directly related to the perception of the level of self-mastery and self-efficacy toward the environment. The last two items come from the self-esteem scale by Rosenberg (1965) and reflect the appraisal of one’s own worth
		The items were averaged separately in 2015 and 2018, with a higher score indicating a greater self-mastery. Subsequently, the overall scores from 2015 and 2018 (at least one answer was required) were averaged and rounded to the closest integer
*Social circumstances (socioeconomic characteristics and perceived social circumstances)*	Years of education	Years of education by 2018 based on the International Standard Classification of Education Classification
	Household income	Household income was equalised to take the size and composition of households into account by converting household income into income of one-person households. To compute equivalised household income, the household income is divided by an equivalence scale. The modified the Organisation for Economic Co-operation and Development (OECD) scale was used, which attributes a weight of 1 to the first adult, a weight of 0.5 to all other household members from 14 years on, and a weight of 0.3 to children up to 14 years. The sum of these weights gives the modified OECD scale. As the income variable was highly positively skewed, it was log-transformed
		The household income between 2013 and 2018 was averaged (at least one measure was required) and rounded to the closest integer
	Socioeconomic prestige	Socioeconomic prestige was measured using the Treiman’s Prestige Scale (Treiman, 1977). The subjectively attributed prestige of a specific occupation is (a) linked to the privilege and power which individuals enjoy based on their occupational titles, (b) invariant across social and cultural groupings, and (c) similar across all complex modern societies. The prestige scores range between 0 (lowest prestige) and 100 (highest prestige)
	Satisfaction with financial situation	The satisfaction with financial situation was measured with a question:
		“Overall how satisfied are you with your financial situation, if 0 means "not at all satisfied" and 10 "completely satisfied"?”
		The satisfaction with financial was averaged between 2013 and 2018 (at least one measure was required) and rounded to the nearest integer
	Job insecurity	Job insecurity was measured with a question “How do you evaluate the risk of becoming personally unemployed in the next 12 months, if 0 means "no risk at all" and 10 "a real risk"?” asked in 2014. The score was averaged between 2013 and 2018 (at least one measure was required) and rounded to the nearest integer
	Job satisfaction	Participants were asked annually between 2013 and 2018: “On a scale from 0 "not at all satisfied" to 10 "completely satisfied" can you indicate your degree of satisfaction for each of the following points?” Work conditions; work atmosphere; interest in tasks; amount of work; hierarchical superiors; promotion; job in general
		The items were averaged for each year separately and then across the years (at least one measure was required). The scores were rounded to the nearest integer
	Job demands	Job demands were measured between 2013 and 2018 using three items: interference between work and private activities/family obligations, being exhausted after work to do what they would like, difficulty to disconnect from work, which were adapted and developed from work of Netemeyer and colleagues (Netemeyer et al., 1996). The response options on each item ranged from 0 “not at all” to 10 “very strongly”. The items were averaged within each year and then the overall scores were averaged between 2013 and 2018 (at least one measure was required). The scores were rounded to the nearest integer, with a higher score indicating greater demands
	Satisfaction with leisure activities	Participants were asked annually between 2013 and 2018 about satisfaction with leisure activities. The response options ranged from 0 “not at all satisfied” to 10 “completely satisfied”. The responses on both items were averaged for each year separately, then across all years (at least one measure was required), and the score was rounded to the nearest integer
	Social support	Participants were asked annually between 2013 and 2018 about the extent to which they receive practical and emotional support from relatives, close friends, and siblings. The response options ranged from 0 “not at all” to 10 “a great deal”. The responses were averaged across the items within each year, then across the years, and rounded to the nearest integer
	Relationships satisfaction	Participants were asked annually between 2013 and 2018 about satisfaction with personal relationships. The response options ranged from 0 “not at all satisfied” to 10 “completely satisfied”. The responses were averaged across the years and rounded to the nearest integer
	Housing satisfaction	Participants rated their housing satisfaction on the scale between 0 “completely dissatisfied” to 10 “completely satisfied”. The responses were averaged across the years (at least one measure was required) and rounded to the nearest integer
*Structural factors (self-perceived factors aggregated at the cantonal level and objectively measured factors)*	Satisfaction with democracy	Participants rated their satisfaction with democracy in 2014 and 2017 on the scale from 0 “not at all satisfied” to 10 “completely satisfied”. The responses were averaged across the years (at least one measure was required). The variable was aggregated on the cantonal level
	Perceived political influence	Participants rated their perception of political influence in 2014 and 2017 on the scale from 0 “no influence” to 10 “a very strong influence”. The responses were averaged across the years (at least one measure was required). The variable was aggregated on the cantonal level
	Trust in people	Participants rated their trust in people annually between 2013 and 2018 on the scale from 0 “can’t be too careful” to 10 “most people can be trusted”
		The responses were averaged across the years (at least one measure was required) and rounded to the nearest integer. The variable was aggregated on the cantonal level
	Trust in Federal Government	Participants rated their trust in the Federal Government in 2014 and 2017 on the scale from 0 “no confidence” to 10 “full confidence”. The responses were averaged across the years (at least one measure was required). The variable was aggregated on the cantonal level
	Gender inequality	Participants rated gender inequality by answering two questions: “Do you have the feeling that in Switzerland women are penalized compared with men in certain areas?” and “Are you in favour of Switzerland taking more steps to ensure the promotion of women?” in 2014 and 2017. The response scale ranged from 0 “not at all penalized” to 10 “strongly penalised”. The responses were averaged across the years (at least one measure was required). The variable was aggregated on the cantonal level
	Unemployment rate	The unemployment rate in 2018 at the cantonal level is equal to the ratio between the number of registered unemployed individuals and the active resident population, calculated as the average of the 2015, 2016 and 2017 structural surveys (SECO - Secrétariat d'Etat à l'économie, 2018)
	Gross Domestic Product	Gross domestic product per capita in Swiss francs in each canton in 2016 (Federal Statistical Office, 2019)
	Home ownership	The estimated figures of homeownership rate from the structural survey in each canton in 2017 (Federal Statistical Office, 2019)
	Physicians per capita	Doctors in the outpatient sector per 100,000 inhabitants in each canton in 2017 (Federal Statistical Office, 2019)
*Mental health/wellbeing*	Life satisfaction	Life satisfaction was measured in 2015 and 2018 using four items: life close to ideal; excellent life conditions; having gotten important things; not changing anything
		The response options ranged from 0 “completely disagree” to 10 “completely agree”. The items were averaged within each year, then the overall scores were averaged across the years (at least one measure was required) and rounded to the nearest integer
	Energy and optimism	Participants were asked annually between 2013 and 2018 “Are you often plenty of strength, energy and optimism, if 0 means "never" and 10 "always"?” The response options were averaged across the years (at least one response was required) and rounded to the nearest integer
	Joy	Participants were asked annually between 2013 and 2018 “How frequently do you generally experience the following emotions, if 0 means "never" and 10 "always"?” The response options were averaged across the years (at least one response was required) and rounded to the nearest integer
	Anger	Participants were asked annually between 2013 and 2018 “How frequently do you generally experience the following emotions, if 0 means "never" and 10 "always"?” The response options were averaged across the years (at least one response was required) and rounded to the nearest integer
	Sadness	Participants were asked annually between 2013 and 2018 “How frequently do you generally experience the following emotions, if 0 means "never" and 10 "always"?” The response options were averaged across the years (at least one response was required) and rounded to the nearest integer
	Worry	Participants were asked annually between 2013 and 2018 “How frequently do you generally experience the following emotions, if 0 means "never" and 10 "always"?” The response options were averaged across the years (at least one response was required) and rounded to the nearest integer

### Age Differences Across Variables

To examine age differences across variables, we regressed each correlate and indicator of mental health and wellbeing on a dummy variable representing the age group (midlife vs young adults as a reference group). The age comparisons are expressed as a mean difference between the groups and the corresponding effect size (Cohen’s d) (Cohen, 1988).

### Network Analysis

**Fig. 1:**
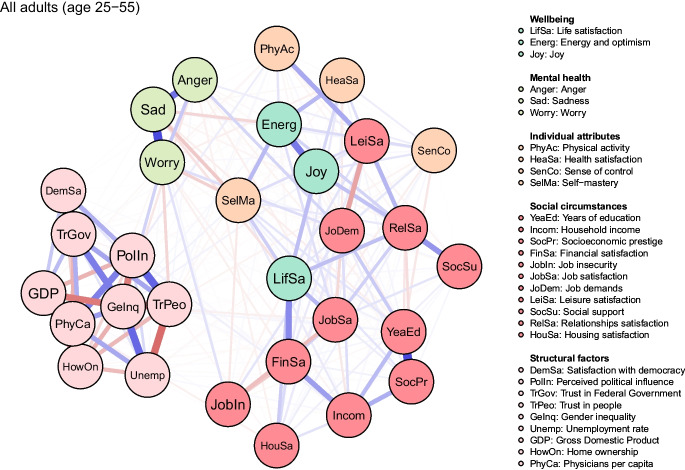
The network model for all participants combined. Legend: blue lines = positive interrelations; red lines = negative interrelations

We fitted three network models, one for all participants combined (25–55 years), one for young (25–39 years) and one for middle-aged adults (40–55 years), capturing regularised partial correlations between continuous variables. These models provide information about the sign (positive vs negative) and size of interrelations between factors after controlling for all other included variables and reducing the number of spurious associations (Epskamp et al., 2018). The network model was visualised (see Fig. 1), with variables depicted as circles (‘nodes’) and the relationships between them illustrated as lines (‘edges’) (Epskamp et al., 2018). The strength of the relationship between two variables is represented by the thickness of the edges (thicker line = stronger relationship), and the direction by the colour (positive = blue, negative = red) (Epskamp & Fried, 2018). To reduce the risk of false-positive interrelations, which may result from estimating many edges, we used the least absolute shrinkage and selection operator (LASSO) regularisation method (Epskamp et al., 2018). This method sets weak partial correlations to zero (using a tuning parameter lambda = 0.5), resulting in more parsimonious and sparser networks. The network structures were estimated using ‘estimateNetwork’ function in R (version 4.1.2), with the extended Bayesian information criterion graphical least absolute shrinkage and selection operator (EBICglasso) default from the ‘qgraph’ package (Epskamp & Fried, 2018; Epskamp et al., 2012). Networks were illustrated using the ‘qgraph’ package (Epskamp et al., 2012), which implements the Fruchterman–Reingold algorithm to place highly connected nodes closer together (Fruchterman & Reingold, 1991).

### Network Stability and Accuracy

To investigate the robustness of the estimated network models, we examined their stability and accuracy (more details can be found in the supplementary material eText 1 and eFigures 1 & 2). Accuracy was assessed by inspecting the width of confidence intervals for the interrelations between variables, calculated using a nonparametric bootstrap method. Stability was tested by comparing the nodes strength in sample subsets, their high similarity would indicate a stable network (Epskamp et al., 2018). We used the ‘bootnet’ package in R (Epskamp et al., 2018), with 2000 bootstraps each to scrutinise stability and accuracy.

### Networks Comparison

First, we tested whether the *overall level of connectivity (or density)* was the same in midlife (40–55 years) and young adulthood (25–39 years) (invariant global strength). The network connectivity was measured as the absolute sum of all edges in the network, with negative edges treated as positive (Opsahl et al., 2010). A denser network (i.e., having stronger global connectivity) may result from stronger interrelations within the correlates, within the indicators, between correlates and indicators of mental health and wellbeing, or combinations of these. For instance, the networks would be denser in midlife (40–55 years) than in young adulthood (25–39 years) if both protective and risk factors for mental health and wellbeing potentially cumulate in midlife, certain correlates having a potentially stronger interrelation with mental health and wellbeing (e.g., physical health), and mental health being worse in this life phase.

Second, we tested whether there were any *differences in the interrelations between each correlate and indicator of mental health and wellbeing.* These differences may result either from the strength of the relationship between a given correlate and indicator or from the difference in the shortest path between these variables. That is, a correlate may have a pathway of equal distance to an indicator (e.g., they are directly connected or via one mediator in both networks), but the strength of the relationship(s) is stronger in one network than the other. Alternatively, the pathway between a correlate and indicator can differ in length, for example, in one network the correlate is linked via one mediator with the indicator and via two in the other network. The length difference, along the strongest connection(s), between variables indicates the ‘quickest’ way to traverse the network between these two variables (Fritz et al., 2018).

The potential differences in the global strength and interrelations between individual edges were tested using the ‘NetworkComparisonTest’ (NCT) R package (van Borkulo et al., 2022). The NCT assess the differences of the networks using resampling-based permutation testing in three steps. First, the relevant test statistics based on the two network models are estimated (i.e., the sum of edge weights and differences in individual edge weights). Second, participants are repeatedly and randomly shuffled between networks and the test statistics are re-estimated. Third, the statistical significance of the differences is determined based on a reference distribution created from the obtained test statistics (van Borkulo et al., 2022). Networks were compared using 1,000 random permutations. We included 24 correlates and 6 indicators of mental health and wellbeing in the network models, which resulted in 144 tests of their interrelations. As the analysis was exploratory, results without correction for multiple testing are presented.

### Missing Data

Missing information in individual variables ranged from less than 1% for self-mastery, financial situation, leisure satisfaction, relationships satisfaction, and housing satisfaction in both age groups to 27.2% for social support in young adulthood and 20.7% for years of education in middle adulthood (see Table 2). Young adults had a greater proportion of missing information in sense of control (16.1 vs 7.2%), social support (27.2 vs 20.0%), and health satisfaction (11.6 vs 4.5%) than middle-aged adults. Middle-aged individuals had a greater proportion of missing data on job satisfaction (18.1 vs 9.9%) than young adults.

**Table 2:** Descriptive information about the study sample – stratified by age groups

	Young adulthood (25–39 years of age)							Middle adulthood (40–55 years of age)						
	N	Missing n	Missing %	Mean	SD	Min	Max	N	Missing n	Missing %	Mean	SD	Min	Max
*Mental health/wellbeing*														
Life satisfaction	2044	0	0.0	7.71	1.28	2	10	3271	0	0.0	7.57	1.30	0	10
Energy and optimism	2044	0	0.0	7.25	1.29	0	10	3271	0	0.0	7.26	1.38	0	10
Joy	2044	0	0.0	7.65	0.95	3	10	3271	0	0.0	7.50	1.03	2	10
Anger	2044	0	0.0	4.13	1.52	0	9	3271	0	0.0	4.26	1.57	0	10
Sadness	2044	0	0.0	3.27	1.48	0	9	3271	0	0.0	3.58	1.56	0	10
Worry	2044	0	0.0	3.14	1.95	0	10	3271	0	0.0	3.36	1.96	0	10
*Individual factors*														
Physical activity	2043	1	0.1	2.51	1.59	0	7	3271	0	0.0	2.66	1.69	0	7
Health satisfaction	1808	236	11.6	8.08	1.61	0	10	3125	146	4.5	7.80	1.73	0	10
Sense of control	1716	328	16.1	5.99	0.92	2	10	3035	236	7.2	5.93	0.97	0	10
Self-mastery	2040	4	0.2	7.64	1.16	4	11	3261	10	0.3	7.68	1.21	3	11
*Social circumstances*														
Years of education	1661	383	18.7	15.02	3.10	8	21	2593	678	20.7	14.51	3.07	8	21
Household income	2011	33	1.6	11.16	0.42	8.16	12.84	3173	98	3.0	11.19	0.47	8.85	13.23
Socioeconomic prestige	1916	128	6.3	47.67	12.15	6	78	3082	189	5.8	46.75	12.57	13	78
Financial satisfaction	2043	1	0.1	6.77	1.81	0	10	3271	0	0.0	7.12	1.78	0	10
Job insecurity	1984	60	2.9	2.07	1.96	0	10	3162	109	3.3	2.28	2.05	0	10
Job satisfaction	1841	203	9.9	7.45	1.12	1	10	2679	592	18.1	7.44	1.17	0	10
Job demands	1987	57	2.8	3.96	1.74	0	9	3166	105	3.2	4.13	1.84	0	10
Leisure satisfaction	2041	3	0.2	7.62	1.46	0	10	3265	6	0.2	7.65	1.54	0	10
Social support	1489	555	27.2	8.00	1.43	1	10	2618	653	20.0	7.40	1.69	0	10
Relationships satisfaction	2042	2	0.1	8.26	1.13	2	10	3271	0	0.0	8.20	1.21	3	10
Housing satisfaction	2044	0	0.0	8.45	1.21	2	10	3271	0	0.0	8.44	1.25	1	10
*Structural factors*														
Satisfaction with democracy	2044	0	0.0	6.45	0.15	4.86	6.96	3271	0	0.0	6.46	0.14	5.45	6.96
Perceived political influence	2044	0	0.0	4.23	0.29	2.45	5.01	3271	0	0.0	4.20	0.30	2.45	5.01
Trust in Federal Government	2044	0	0.0	6.01	0.17	5.14	6.53	3271	0	0.0	6.03	0.16	5.16	6.60
Trust in people	2044	0	0.0	6.26	0.39	4.43	7.40	3271	0	0.0	6.22	0.40	4.25	7.39
Gender inequality	2044	0	0.0	11.07	1.13	4.57	13.38	3271	0	0.0	11.20	1.16	6.51	13.38
Unemployment rate	1807	237	11.6	3.31	1.11	0.91	5.55	3157	114	3.5	3.38	1.11	0.91	5.55
Gross Domestic Product	1807	237	11.6	77560.36	21543.62	51198.58	165604.91	3157	114	3.5	76924.60	20458.93	51198.58	165604.91
Home ownership	1807	237	11.6	37.44	9.36	15.30	57.40	3157	114	3.5	37.98	9.55	15.30	57.40
Physicians per capita	1807	237	11.6	212.32	61.08	91.61	424.63	3157	114	3.5	212.28	59.79	91.61	424.63

Those with missing information on any variable were more likely to be men, non-Swiss, speak French or Italian (rather than German), have lower life satisfaction, fewer years of education, lower income, lower social support, and trust in people (see eTable 1). Among young adults, trust in people was more strongly associated with the likelihood of missing information than in middle-aged individuals.

Missing data were imputed using the R package ‘missForest’, which uses an iterative imputation method based on random forests (Stekhoven & Buhlmann, 2012). This non-parametric approach was found to be highly accurate, outperforming nearest neighbour imputation and multivariate imputation by chained equations in simulated missing data (Waljee et al., 2013).

### Post-hoc Analysis: Correcting for Life Stressors

As a post-hoc analysis, we examined life stressors as a potential source of observed age differences between the networks. Life stressors are defined as significant occurrences, often with far-reaching consequences that require individuals to adjust to changing circumstances (Luhmann et al., 2012). These can occur at any age but are more likely to accumulate in midlife (Comolli et al., Under review), and may potentially have more severe consequences in this life phase. In our study, they include illness or accident, death of a closely related person, termination of a close relationship, problems with children, caring responsibilities for children, caring responsibilities for the elderly, job loss, and divorce. Details on how they were measured can be found in Table 3. We reran the age-stratified networks while correcting for the count of life stressors (ranging 0–8).

**Table 3:** Details about measurement of the life stressors

Illness/accident; death of closely related person; termination of close relationship; problems with own children, divorce and unemployment	Participants reported, annually between 2013 and 2018, experiencing any of the following since the previous interview: Illness/accident; death of a closely related person; termination of close relationship; problems with own children, divorce and unemployment
Caring responsibilities for elderly	Respondents were asked in the household survey, annually between 2014 and 2016, whether they or another person in the household were caring for elderly within the household or for parents, or parents-in-law outside of the household. An affirmative answer was counted as caring responsibility
Caring responsibilities for children	Respondents were asked in the household survey, annually between 2014 and 2016, whether they or another person in the household were caring for child(ren) within or outside of the household. An affirmative answer was counted as caring responsibility. In addition, they were asked whether they or their partner were responsible for childcare including monthly expenses, caring in case of illness, playing with child(ren), taking them to kindergarten or school, or helping with homework
Deriving the overall variable of life stressors	First, we derived an indicator of experiencing any of the above events within the observation period. Each participant could experience one occurrence within each category of events, including illness or accident, death of a closely related person, termination of a close relationship, problems with children, caring responsibilities for children, caring responsibilities for elderly, job loss, and divorce. Secondly, we summed up the total number of events within the observation period, resulting in a potential range of 0–8

## Results

### Age Differences Across Variables

Descriptive information about all study variables can be found in Table 2 and age differences in Table 4. Middle-aged individuals had worse mental health and wellbeing on all indicators but energy and optimism, which did not differ across groups. However, the effect sizes (according to Cohen’s d) were small, reaching the maximum of 0.20 for sadness.

**Table 4:** Age differences between young and middle-aged adults in all study variables – expressed as a mean difference and the corresponding effect size (Cohen’s d or standardised mean difference)

	mean difference	CI 95%	Cohen's d	CI 95%
*Mental health/wellbeing*				
Life satisfaction	-0.14	(-0.21, -0.07)	0.11	(0.17, -0.06)
Energy and optimism	0.01	(-0.06, 0.09)	0.01	(-0.05, 0.06)
Joy	-0.15	(-0.21, -0.10)	0.15	(0.21, -0.09)
Anger	0.14	(0.05, 0.22)	0.09	(0.03, 0.14)
Sadness	0.31	(0.22, 0.39)	0.20	(0.15, 0.26)
Worry	0.22	(0.11, 0.33)	0.11	(0.06, 0.17)
*Individual factors*				
Physical activity	0.16	(0.06, 0.25)	0.09	(0.04, 0.15)
Health satisfaction	-0.27	(-0.37, -0.18)	0.16	(0.22, 0.10)
Sense of control	-0.06	(-0.12, -0.00)	0.06	(0.12, 0.00)
Self-mastery	0.03	(-0.03, 0.10)	0.03	(-0.03, 0.08)
*Social circumstances*				
Years of education	-0.52	(-0.71, -0.33)	0.17	(0.23, 0.11)
Household income (log transformed)	0.04	(0.01, 0.06)	0.09	(0.03, 0.14)
Socioeconomic prestige	-0.92	(-1.63, -0.22)	0.07	(0.13, 0.02)
Financial satisfaction	0.35	(0.25, 0.45)	0.19	(0.14, 0.25)
Job insecurity	0.21	(0.10, 0.33)	0.11	(0.05, 0.16)
Job satisfaction	-0.01	(-0.08, 0.05)	0.01	(-0.07, 0.05)
Job demands	0.17	(0.07, 0.27)	0.09	(0.04, 0.15)
Leisure satisfaction	0.03	(-0.06, 0.11)	0.02	(-0.04, 0.07)
Social support	-0.59	(-0.69, -0.49)	0.37	(0.43, 0.31)
Relationships satisfaction	-0.06	(-0.13, 0.01)	0.05	(0.11, 0.00)
Housing satisfaction	-0.02	(-0.08, 0.05)	0.01	(-0.07, 0.04)
*Structural factors*				
Satisfaction with democracy	0.01	(-0.00, 0.02)	0.05	(-0.01, 0.10)
Perceived political influence	-0.03	(-0.10, -0.01)	0.10	(-0.15, -0.04)
Trust in Federal Government	0.02	(0.01, 0.03)	0.12	(0.06, 0.17)
Trust in people	-0.04	(-0.06, -0.01)	0.09	(-0.15, -0.04)
Gender inequality	0.14	(0.07, 0.20)	0.12	(0.06, 0.17)
Unemployment rate	0.08	(0.01, 0.14)	0.07	(0.01, 0.13)
Gross Domestic Product	-635.8	(-1842.1, 570.6)	0.03	(-0.09, 0.03)
Home ownership	0.54	(-0.00, 1.09)	0.06	(0.00, 0.12)
Physicians per capita	-0.04	(-3.53, 3.44)	0.00	(-0.06, 0.06)

Among individual factors, middle-aged participants had lower health satisfaction despite exercising more and had a lower sense of control. Some measures of social circumstances also varied between age groups, with household income and satisfaction with the financial situation being higher in midlife, and job insecurity, job demands, and socioeconomic prestige being lower. Both perceived social support and relationships satisfaction were lower in midlife. The effect sizes of these differences reached the maximum of 0.37 for social support. In terms of structural factors, there were marginally higher values among midlife individuals in trust in the Federal Government, gender inequality, and unemployment rate. Perceived political influence and trust in people were marginally lower in the midlife subgroup. As structural factors were aggregated at the cantonal level, they represent differences in the age structure of the Swiss cantons rather than (averaged) individual differences. For instance, a higher unemployment rate in midlife group means that they are more likely to live in the cantons with higher unemployment.

### Network Analysis Across all Participants

The network model for all participants was highly dense, comprising 307 (70.3%) edges of potential 437 estimated to be above zero (see Fig. 1). The network had high accuracy, as indicated by an acceptable width of the bootstrapped confidence intervals around edges (see eFigure 1). The model was also characterised by a high node strength stability, as we found that up to 74.9 per cent of the sample could be dropped to detect an association of at least 0.7 between the subset and the original node strength coefficients (see eFigure 2).

From visually inspecting the network model, we can see that mental health factors cluster closely together, with sadness connecting both worry and anger. In terms of wellbeing, energy and optimism is strongly correlated with joy. Life satisfaction appears to be bridging various clusters of social variables. It is simultaneously linked with a cluster of socioeconomic correlates, including income and financial satisfaction, as well as, with indicators of leisure and relationship satisfaction, which were closely interrelated. Individual factors did not appear to cluster together. Salf-mastery seems to interlink mental health and wellbeing clusters. Physical activity is closely related to job and socioeconomic circumstances. Finally, structural factors formed tight clusters, probably as they were aggregated at the cantonal level, as opposed to being measured at the level of an individual as other variables. They were interrelated mainly with worry, through trust in people and unemployment.

Alternatively, there may be clusters (e.g., various indicators of socioeconomic circumstances) or chains of variables (e.g., due to spill-over effects across different life domains) that are linked with mental health and wellbeing.

Non-zero (up to two decimal points) correlates of mental health and wellbeing can be found in Table 5, in the order of the strongest to the weakest regardless of the direction of the relationship. The most consistent correlates across all three indicators of wellbeing (life satisfaction, joy, energy and optimism) were job and relationships satisfaction. The strongest correlates of overall life satisfaction were financial situation (edge weight; EW = 0.28), job satisfaction (EW = 0.17), relationships satisfaction (EW = 0.15), housing satisfaction (EW = 0.12), self-mastery (EW = 0.12), health satisfaction (EW = 0.10), leisure satisfaction (EW = 0.07), and sense of control (EW = 0.05). Joy was most strongly correlated with relationships satisfaction (EW = 0.15), social support (EW = 0.07), sense of control (EW = 0.06), and leisure satisfaction (EW = 0.05). Energy and optimism, in turn, had the strongest relationship with health satisfaction (EW = 0.17), self-mastery (EW = 0.17), relationships satisfaction (EW = 0.11), sense of control (EW = 0.08), and job satisfaction (EW = 0.07).

**Table 5:** Nonzero-edges in either age group (or combined) connected to indicators of mental health and wellbeing

Path			Edge weight		
Correlate		Indicator	T	Y	M
Financial situation	↔	Life satisfaction	0.28	0.27	0.29
Health satisfaction	↔	Energy and optimism	0.17	**0.12**	**0.21**
Job satisfaction	↔	Life satisfaction	0.17	0.15	0.19
Self-mastery	↔	Energy and optimism	0.17	0.18	0.15
Relationships satisfaction	↔	Life satisfaction	0.15	0.11	0.17
Relationships satisfaction	↔	Joy	0.15	0.16	0.15
Self-mastery	↔	Sadness	-0.13	-0.14	-0.13
Housing satisfaction	↔	Life satisfaction	0.12	**0.08**	**0.14**
Self-mastery	↔	Life satisfaction	0.12	0.13	0.13
Gender inequality	↔	Worry	0.11	0.12	0.11
Self-mastery	↔	Worry	-0.11	-0.06	-0.13
Relationships satisfaction	↔	Energy and optimism	0.11	0.12	0.10
Trust in people	↔	Worry	-0.10	**-0.12**	**-0.09**
Job demands	↔	Anger	0.10	0.07	0.10
Health satisfaction	↔	Life satisfaction	0.10	0.08	0.10
Sense of control	↔	Energy and optimism	0.08	0.06	0.09
Job demands	↔	Worry	0.08	0.06	0.09
Job satisfaction	↔	Energy and optimism	0.07	0.08	0.06
Leisure satisfaction	↔	Life satisfaction	0.07	0.08	0.07
Job insecurity	↔	Worry	0.07	0.06	0.08
Social support	↔	Joy	0.07	0.04	0.05
Sense of control	↔	Joy	0.06	0.07	0.05
Gender inequality	↔	Anger	-0.06	-0.05	-0.06
Leisure satisfaction	↔	Joy	0.05	0.03	0.07
Sense of control	↔	Life satisfaction	0.05	0.02	0.05
Job satisfaction	↔	Joy	0.04	0.03	0.06
Physical activity	↔	Energy and optimism	0.04	0.07	0.01
Socioeconomic prestige	↔	Life satisfaction	0.04	0.04	0.03
Health satisfaction	↔	Worry	-0.04	-0.04	-0.03
Sense of control	↔	Anger	0.04	0.01	0.05
Years of education	↔	Joy	-0.04	-0.05	-0.03
Financial situation	↔	Worry	-0.04	-0.06	-0.03
Years of education	↔	Sadness	-0.04	-0.02	-0.03
Health satisfaction	↔	Joy	0.04	0.05	0.01
Physical activity	↔	Life satisfaction	-0.03	-0.01	-0.03
Job insecurity	↔	Life satisfaction	-0.03	0.00	-0.05
Years of education	↔	Anger	-0.03	-0.06	-0.01
Housing satisfaction	↔	Anger	-0.03	-0.03	-0.03
Job satisfaction	↔	Anger	-0.03	0.00	-0.04
Years of education	↔	Life satisfaction	0.03	0.04	0.01
Sense of control	↔	Sadness	0.03	0.02	0.03
Job demands	↔	Joy	-0.03	-0.04	0.00
Household income	↔	Worry	-0.02	-0.01	-0.02
Leisure satisfaction	↔	Energy and optimism	0.02	0.02	0.03
Job demands	↔	Energy and optimism	-0.02	-0.01	-0.04
Trust in people	↔	Energy and optimism	-0.02	-0.03	-0.01
Job insecurity	↔	Sadness	0.02	0.02	0.01
Social support	↔	Worry	0.02	0.00	0.02
Years of education	↔	Worry	-0.02	0.00	-0.03
Social support	↔	Life satisfaction	0.02	0.03	0.00
Health satisfaction	↔	Sadness	-0.02	-0.02	-0.01
Relationships satisfaction	↔	Anger	-0.02	-0.01	-0.01
Gender inequality	↔	Energy and optimism	0.02	0.00	0.03
Trust in people	↔	Anger	0.02	0.01	0.01
Sense of control	↔	Worry	0.02	0.01	0.02
Relationships satisfaction	↔	Sadness	-0.02	-0.01	-0.02
Self-mastery	↔	Anger	-0.01	-0.02	-0.02
Self-mastery	↔	Joy	0.01	0.00	0.02
Financial situation	↔	Joy	-0.01	0.00	0.00
Household income	↔	Life satisfaction	0.01	0.02	0.01
Unemployment rate	↔	Worry	0.01	0.01	0.02
Household income	↔	Joy	-0.01	0.00	-0.02
Home ownership	↔	Worry	-0.01	0.00	-0.01
Socioeconomic prestige	↔	Energy and optimism	-0.01	0.00	0.00
Physical activity	↔	Anger	0.01	0.02	0.00
Leisure satisfaction	↔	Sadness	0.01	0.00	0.01
Gross Domestic Product	↔	Worry	-0.01	0.00	-0.01
Job insecurity	↔	Energy and optimism	0.01	0.00	0.01
Socioeconomic prestige	↔	Anger	0.00	-0.01	0.00
Perceived political influence	↔	Joy	0.00	-0.01	0.00
Social support	↔	Energy and optimism	0.00	0.01	0.01
Trust in people	↔	Joy	0.00	-0.01	0.00
Financial situation	↔	Anger	0.00	-0.03	0.00
Gross Domestic Product	↔	Anger	0.00	0.00	0.01
Physicians per capita	↔	Life satisfaction	0.00	-0.01	0.00
Household income	↔	Sadness	0.00	0.00	-0.02
Socioeconomic prestige	↔	Sadness	0.00	0.00	-0.01
Financial situation	↔	Energy and optimism	0.00	0.00	-0.01
Financial situation	↔	Sadness	0.00	0.00	-0.01
Job insecurity	↔	Joy	0.00	-0.03	0.02
Job demands	↔	Sadness	0.00	0.04	0.00
Social support	↔	Sadness	0.00	0.00	0.01
Housing satisfaction	↔	Joy	0.00	0.00	0.02
Housing satisfaction	↔	Sadness	0.00	-0.02	0.00
Housing satisfaction	↔	Worry	0.00	0.00	0.02
Satisfaction with democracy	↔	Energy and optimism	0.00	-0.01	0.00
Trust in Federal Government	↔	Anger	0.00	-0.01	0.00
Trust in Federal Government	↔	Sadness	0.00	-0.02	0.00
Unemployment rate	↔	Anger	0.00	-0.02	0.00
Physicians per capita	↔	Sadness	0.00	0.01	0.00

T = total sample; Y = young adults (age 25–39); M = middle-aged adults (age 40–55)

Note. We only included non-zero edges that were greater than zero after rounding to the second decimal place. Some edges that were marginally greater than zero were not included

Edge weights in bold represent edges that differed across age groups

The key correlates of mental health differed somewhat from those identified for wellbeing, with self-mastery and job demands being most consistently associated across worry, sadness, anger. Self-mastery also appeared to bridge the indicators of mental health and wellbeing. The strongest correlates of worry were gender inequality (EW = 0.11), self-mastery (EW = -0.11), trust in people (EW = -0.10), job demands (EW = 0.08), and job insecurity (EW = 0.07). Sadness was most strongly linked with self-mastery (EW = -0.13), anger was associated with job demands (EW = 0.10).

### Networks Comparison: Midlife versus Younger Adults

**Fig. 2:**
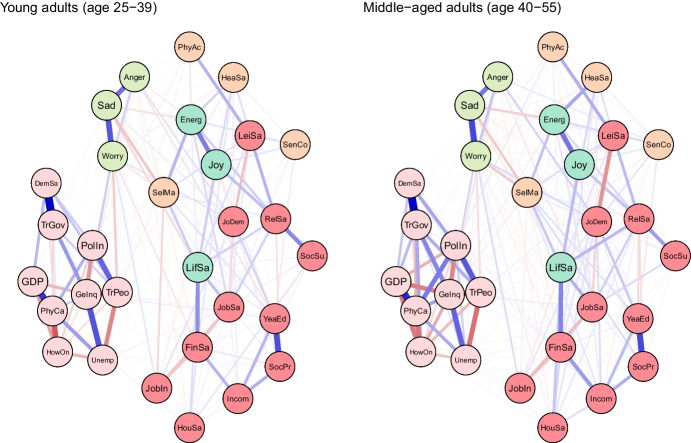
The network models for young and middle-aged adults. Legend: blue lines = positive interrelations; red lines = negative interrelations

**Fig. 3:**
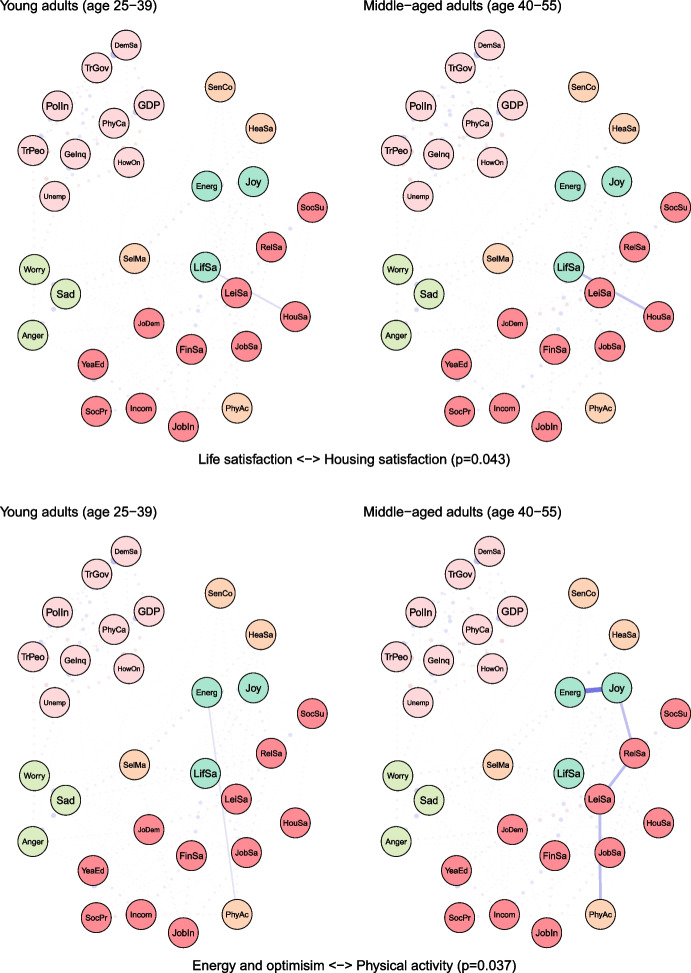

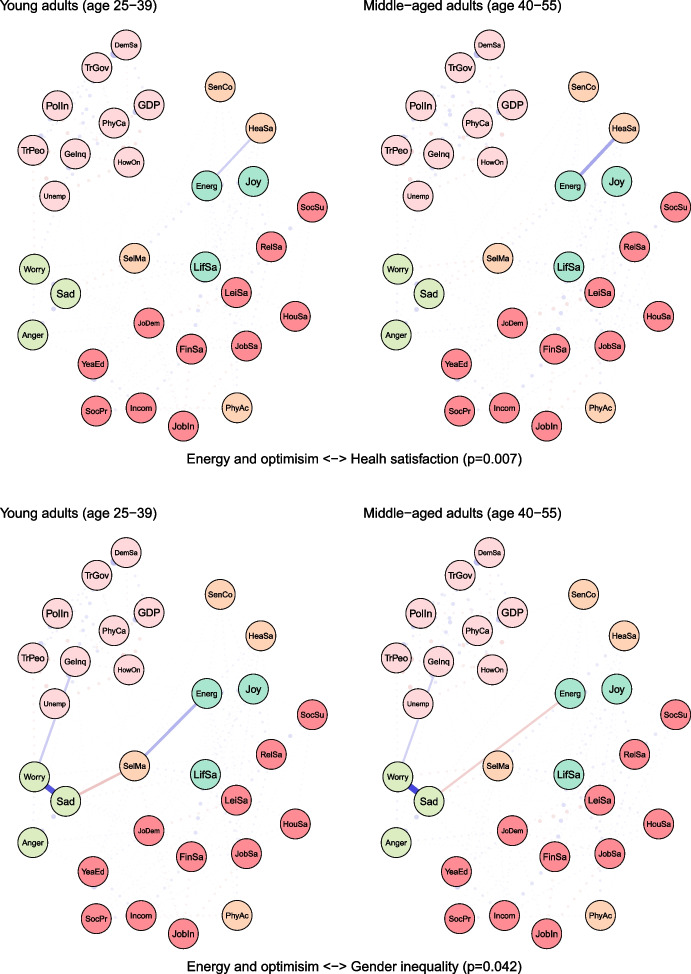

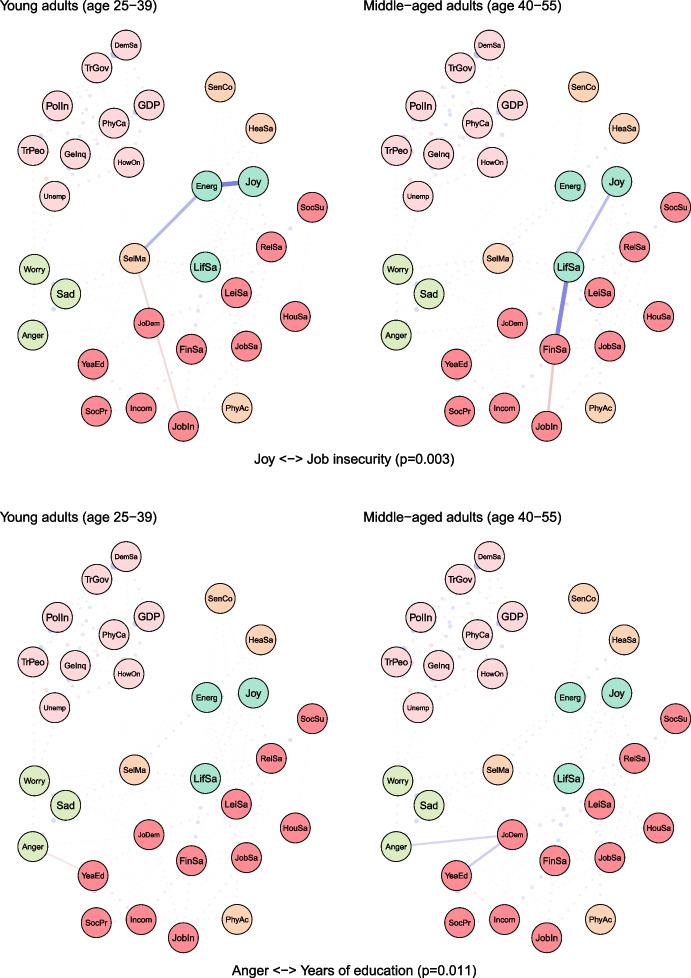

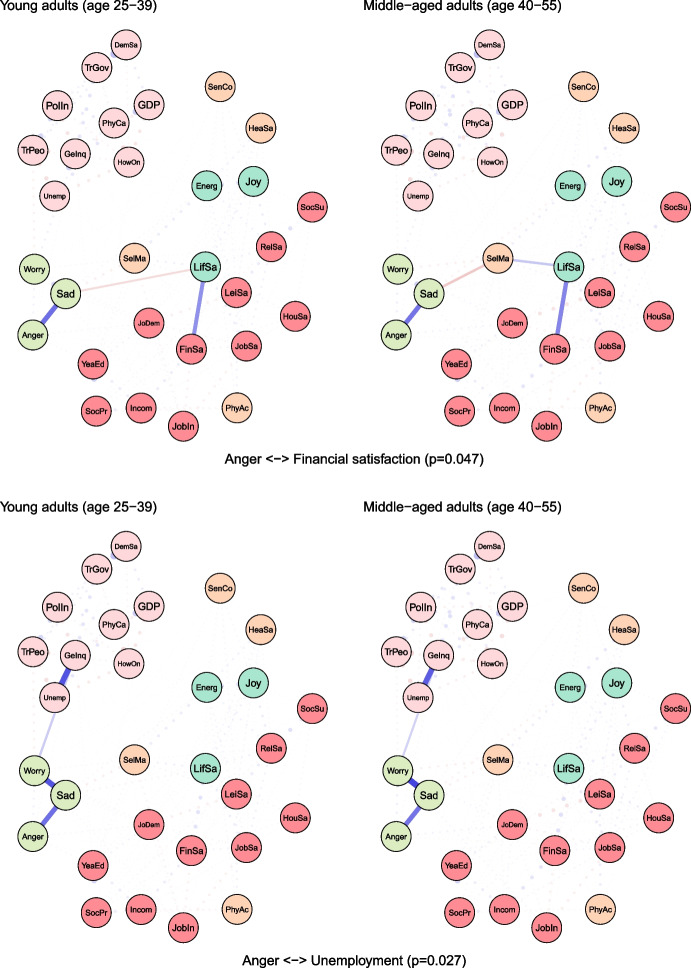

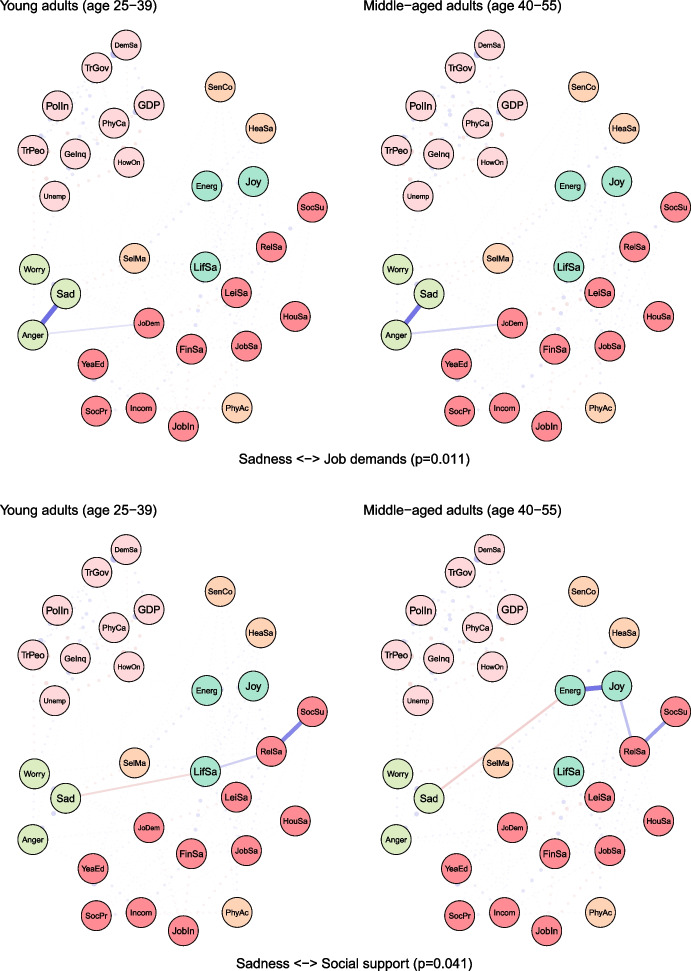

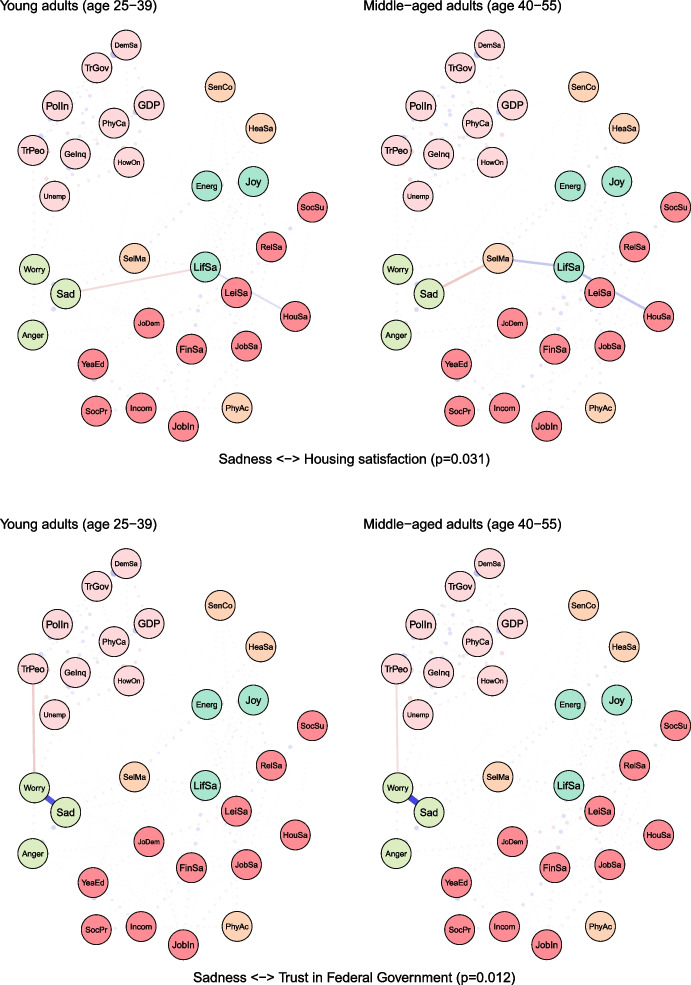

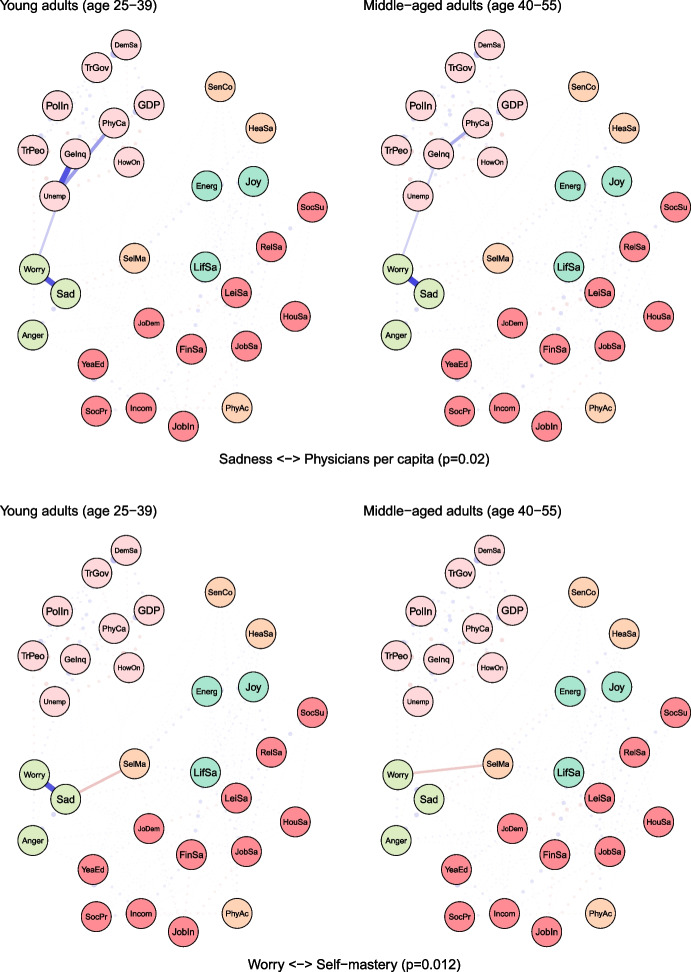

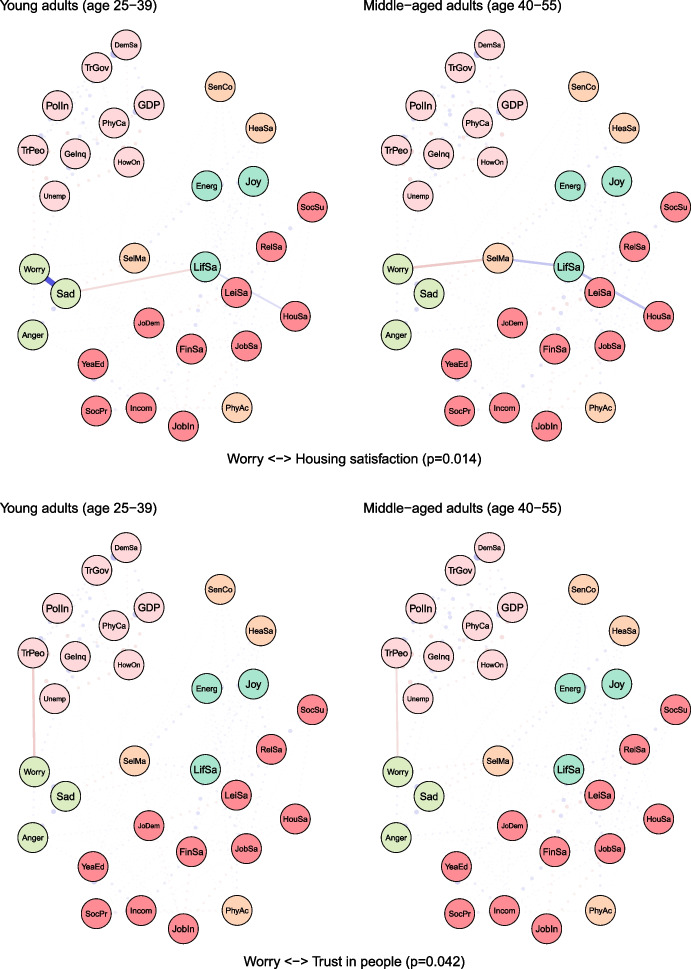
Differing pathways between young and middle-aged adults. Legend: non-transparent, continuous blue lines = positive interrelations; non-transparent, continuous red lines = negative interrelations; transparent, dotted lines = all remaining partial regularized correlation relationships

To examine age differences, we compared network models for young (25–39 years) and middle-aged (40–55 years) adults (see Fig. 2). Both networks were characterised by high accuracy and stability (see eFigures 1 & 2). The global strength (or density) was greater in the network of middle-aged individuals than in one of the young adults (17.37 vs 15.36, *p* = 0.048). We also found that the weights were stronger in midlife for health satisfaction ↔ energy and optimism (0.21 vs 0.12, *p* = 0.007), housing satisfaction ↔ life satisfaction (0.14 vs 0.08, *p* = 0.04), trust in people ↔ worry (-0.12 vs -0.09, *p* = 0.042) (see Fig. 3).

Moreover, we identified differences in 13 paths between correlates and indicators of mental health and wellbeing, which either differed in the length of these paths (i.e., the number of edges) or their overall strength (i.e., the sum of edges):

energy and optimism ↔ physical activity (*p* = 0.037), energy and optimism ↔ gender inequality (*p* = 0.042), joy ↔ job insecurity (*p* = 0.003), anger ↔ years of education (*p* = 0.011), anger ↔ financial situation (*p* = 0.047), anger ↔ unemployment (*p* = 0.027), sadness ↔ job demands (*p* = 0.011), sadness ↔ social support (*p* = 0.041), sadness ↔ housing satisfaction (*p* = 0.031), sadness ↔ trust in Federal Government (*p* = 0.012), sadness ↔ physicians per capita (*p* = 0.012), worry ↔ self-mastery (*p* = 0.012), worry ↔ housing satisfaction (*p* = 0.014) (see Fig. 3).

For instance, physical activity and energy seemed to have a weak direct (and positive) correlation among young adults, while in middle-aged individuals these variables were linked indirectly via leisure and relationships satisfaction, joy and energy (see Fig. 3). The shortest pathways between socioeconomic indicators, such as satisfaction with housing or finances, and indicators of mental health led through life satisfaction in both age groups. However, in middle-aged adults, this relationship was additionally bridged by self-mastery. A differential relationship was also found between social support and worry. In young adults, this correlation was interlinked by energy and self-mastery, whereas in middle-aged adults it was mediated by leisure satisfaction and job demands.

### Post-hoc Analysis: Correcting for Life Stressors

Life stressors were more prevalent in midlife than young adulthood (midlife: mean = 2.70, standard deviation = 1.36, median = 3, interquartile range = 2–4 vs young adulthood: mean = 2.52, standard deviation = 1.27, median = 2, interquartile range = 2–3). After correcting for life stressors, the difference in density between networks obtained from middle and young adulthood was no longer reaching statistical significance (corrected models: difference in density = 2.30, *p* = 0.069 vs main models: difference in density = 2.01, *p* = 0.048). The corrected age-stratified models can be found in eFigure 3. We found differences in three paths between correlates and indicators of mental health and wellbeing, which differed in length of these paths (i.e., the number of edges): life satisfaction ↔ life stressors (*p* = 0.045), anger ↔ life stressors (*p* = 0.003), worry ↔ life stressors (*p* = 0.041) (see eFigure 4). In case of anger and worry, life stressors were directly linked with mental health among midlife individuals, whereas this relationship was mediated by income and financial satisfaction among young adults.

## Discussion

### Main Findings

Middle-aged individuals had worse mental health and wellbeing on all indicators but energy and optimism than young adults, which did not differ across groups. Higher household income and financial satisfaction in midlife were reported alongside greater perceived job insecurity and job demands, with socioeconomic prestige being lower. Moreover, middle-aged individuals had lower social support, relationships satisfaction, and health satisfaction (despite exercising more).

The network was marginally denser in midlife, with two direct interrelations being stronger in this age group, where health satisfaction was positively linked with energy and optimism, and housing satisfaction with life satisfaction. There were also several other differences in indirect interrelations between correlates and indicators of mental health and wellbeing. For instance, physical activity and energy and optimism seemed to have a weak direct (and positive) correlation among young adults, while among middle-aged individuals these variables were linked indirectly via leisure and relationships satisfaction, and joy. Life stressors, such as illness, divorce, or caring responsibilities appeared to be more directly linked with mental health among midlife individuals, whereas this relationship was mediated by socioeconomic circumstances among young adults.

### Context and Implications of the Key Findings

Consistently with multiple previous studies in both Swiss and international contexts (Barbuscia & Comolli, 2021; Blanchflower, 2021; Gondek et al., 2021a, 2021b), we found worse mental health and lower wellbeing among middle-aged than young adults. The effect sizes in our study were comparable to those found in public health interventions (Matthay et al., 2021). Improving wellbeing of middle-aged individuals would potentially translate into large benefits at the population level, such as better quality of life or productivity, as they constitute 1.8 million people in Switzerland or one-fifth of the population (population.pyramid.net, 2022), and one-third of Swiss workers are over the age of 50 (swissinfo.ch, 2021). On of the implications of our findings is the need to raise public awareness that wellbeing tends to be lower in midlife. This could reassure middle-aged individuals that they are not isolated in their experiences of lower life satisfaction or higher worry. Moreover, if indeed, decline in wellbeing is an age effect, relatively universal across generations – younger individuals could anticipate and prepare for the elevated risk of lower wellbeing in the near future. Our study also emphasises the importance of monitoring wellbeing, as well as various quality of life indicators, at the population level, which can help to allocate resources more effectively.

#### Social support and relationships satisfaction were lower in midlife.

This is consistent with similar analysis of German panel data, which showed a declining satisfaction with social contacts and leisure time among middle-aged individuals (Otterbach et al., 2018). Job insecurity and job demands were elevated among middle-aged individuals, in contrast with higher income and financial satisfaction in this age group. Middle-aged workers may feel less secure in their employment due to potentially lower prospects of reemployment (swissinfo.ch, 2015), combined with greater financial responsibilities. This is despite a strongly favourable socioeconomic context of Switzerland. Hence, there is a need to encourage greater job security among middle-aged employees and facilitate re-entering the labour market after job loss. This can be achieved, for instance, by a greater provision of adult training. Health satisfaction was lower in midlife, despite a greater frequency of physical activity, which is to be expected due to a large amount of evidence showing that the decline of physical, particularly chronic, health accelerates in midlife.

Consistently with previous studies (Aichele et al., 2019; Handing et al., 2022), social support, social relationships and physical health were strong correlates of mental health and wellbeing both in early and middle adulthood. Moreover, we found socioeconomic circumstances may be particularly important correlates of wellbeing, with housing satisfaction more predictive in midlife. In addition, rarely examined structural factors (Dykxhoorn et al., 2022), such as perceived gender inequality, or trust in people aggregated at the macro-level might be important correlates of worry. Finally, self-mastery, which refers to believe that one’s destiny is controlled by oneself, was a strong predictor both of wellbeing and mental health across both age groups in our study. We found overall little difference in predictive strength of factors across young and middle adulthood. From a public health perspective, this implies that screening for the above factors may help to identify subgroups of populations at risk in either age group. The main contribution of our study in this context is the inclusion of various mental health and wellbeing indicators and a range of structural variables. Future research would benefit from explicitly examining predictors of intraindividual change in mental health and wellbeing between young and middle adulthood to understand who is at the greatest risk to deteriorate between these life phases.

The key objective of this paper was to generate hypotheses regarding mechanisms (or explanatory factors) that may potentially explain the association between age (young vs middle adulthood) and mental health and wellbeing. We used network analysis that has rarely been applied in the context of determinants of mental health or wellbeing, which as shown by our study, can be a valuable tool for data exploration and theory building, particularly due to its powerful visualisation attributes. Based on our findings, we propose self-perceived physical health, social relationships, including social support, and job demands as such potential explanatory mechanisms. These factors are typically considered as modifiable and relatively easy to screen, hence they are potentially actionable by public health interventions. The network model revealed other interesting patterns, for instance, a potential role of self-mastery in bridging mental health and wellbeing and sadness linking feelings of anger and worry.

### Self-Perceived Physical Health

In our study, health satisfaction was not only lower in midlife than in young adulthood, but it was also more strongly interrelated with energy and optimism in this age group, which in turn, was strongly linked with joy. This suggests that the relationship between perceived health and wellbeing may be of greater importance in midlife. As physical health exponentially deteriorates while mental health and wellbeing tend to improve after midlife (Gondek et al., 2021a, 2021b), physical health can be more important for the difference in mental health between young and middle adulthood, than for older age. Possibly, expectations or perceptions of health can play an important role, with middle-aged individuals realising that their health is not as good as it used to be, whereas older adults becoming more accepting of their health getting poorer. Assuming that physical health was found to partially explain the decline in mental health and wellbeing in midlife, public mental health could benefit from health promotion. This could include encouraging, and more importantly facilitating a healthy lifestyle, as physical activity appears to be closely linked with job and socioeconomic circumstances according to our network model. Even small individual-level effects could translate to large population health benefits if the intervention was widely implemented (Matthay et al., 2021). Moreover, physical activity appears to be strongly linked with leisure and relationship satisfaction among middle-aged adults in our study, hence active lifestyle may have cross-over effects on these life aspects. There is some evidence from intervention studies suggesting that better health and exercising are causally linked to mental health across adulthood (Hu et al., 2020), however, no previous study has explicitly examined whether they can explain worse mental health in midlife compared with younger adults.

### Social Relationships and Social Support

We found that relationships satisfaction and social support were much lower in midlife, while they were the most consistent correlates across all three indicators of wellbeing – life satisfaction, energy and optimism, and joy – both among young and middle-aged adults. The importance of social relationships and social support for mental health or wellbeing across the entire lifespan is well-documented (Umberson & Montez, 2010). For instance, a recent study using Mendelian randomisation produced supportive evidence for a causal link between confiding in others and depression, in an age-adjusted analysis of a large biobank database in the United Kingdom (Choi et al., 2020). The satisfaction with social relationships, and to an even greater extent, social support appears to be lower in midlife than in young adulthood, which again, might suggest that these can be the mechanisms partially translating age differences between young and middle adulthood into lower wellbeing. In middle-aged adults, the relationship between social support and worry was bridged by leisure satisfaction and job demands, hence reducing demands and improving leisure satisfaction can have a spillover effect on social support (and vice versa).

### Job Demands

Another factor that might be considered as one of the mechanisms explaining age differences in mental health and wellbeing is job demands, as it was higher in midlife and more strongly linked with anger and sadness in this age group. Moreover, job demands were found to be on the pathway between variables related to social relationships and worry only in middle-aged adults, meaning that reducing job demands could have cross-over effects on both life domains. High job demands (combined with low control) were associated with subsequent depression in multiple longitudinal studies (Madsen et al., 2017). High job demands might lead to a particularly high investment of resources into the workplace, leading to conflict with other life domains, such as social relationships or leisure time (Knecht & Freund, 2016). Our findings suggest that the workplace could provide the space for the efforts to improve mental health and wellbeing in midlife (Kangasniemi et al., 2019), particularly with job satisfaction being one of the strongest correlates of life satisfaction in both age groups.

### Quality of life

The interrelations between negative work experiences and mental health and wellbeing might be exacerbated by higher job insecurity in midlife and additional life stressors, such as caring responsibilities, divorce, illness or accidents, which tend to accumulate to a greater extent in midlife (Office fédéral de la statistique (OFS), 2021; Comolli et al., Under review). Therefore, employers should strive to provide the means of dealing with elevated job demands, while juggling multiple responsibilities and facing other life stressors. This can be done by allowing more flexible work patterns, and adequate leave for reasons related to illness or caring for family members, which can help to integrate various life domains, potentially leading to better mental health and wellbeing (Knecht & Freund, 2016). Some positive changes have been observed, with Switzerland expanding paid leave for caregivers in 2021 (Secrétariat d'Etat à l'économie (SECO), 2022).

### Limitations

It is important to acknowledge that relationships uncovered in the network analysis are not necessarily causal, but it is rather a descriptive approach, potentially useful for hypotheses generation. The associations we are describing are cross-sectional, hence, for instance, we cannot determine the directionality of the associations between the correlates and indicators of mental health and wellbeing. On a related note, the age comparison of the distribution of mental health and wellbeing and their correlates refers to differences between generations as well as age groups. That is, age and cohort effects are confounded, and the findings cannot be attributed to either of these effects with confidence. In our study, we focus mainly on the potential age effects, as the drop in mental health and wellbeing between young and middle adulthood was previously found within the same individuals in longitudinal studies and across different generations (e.g., among born in 1946, 1958 and 1970 in the UK) (Gondek et al., 2021a, 2021b). However, cohort (or generational) effects may play an important role. For instance, middle-aged individuals today may perceive greater job insecurity and higher job demands due to potentially having experienced more stable employment throughout their young adulthood. Future research could examine proposed explanatory factors using datasets with a follow-up of individuals from young to middle adulthood, for instance, the British birth cohorts (Power & Elliott, 2006; Wadsworth et al., 2005). This could be done with a causal mediation analysis, where mental health (or wellbeing) in young adulthood would constitute the exposure, explanatory factors would be the mediators, and mental health (or wellbeing in midlife) would be the outcome. We included more detailed about conducting such a study, along with a directed acyclic graph (DAG) in eText 2.

Another methodological issue to consider is that normative standards for included measures of mental health and wellbeing have not been developed, hence making the evaluation of the effect sizes in the study difficult. Moreover, these measures were self-reported and do not capture a long-term mental health state, based on a specific clinical diagnosis, which means that they may be affected by the temporary mood of the participants. However, we do not suspect any age-related systematic biases in reporting, meaning that age comparisons could be considered legitimate, and the self-reporting nature of the variables is not necessarily a limitation. We did not aim to provide a population-representative epidemiological description of mental health, for which clinical assessments may be desirable.

As one of the assumptions of the network analysis used in our study is a normal distribution of included variables, we excluded several categorical or (zero-inflated) count correlates that are potentially relevant and available in the data, such as marital status, home ownership, or participation in volunteering activities. This was a pragmatic decision, dictated by the fact that approaches allowing for skewed or categorical variables, such as the Mixed Graphical Models, do not provide a measure of network density and direction of the association for categorical variables (Haslbeck & Waldorp, 2020).

The key strength of our study is that it uses large population-based data, broadly representative of the Swiss population, complementing findings from similar studies that included participants from the socioeconomically disadvantaged region of the United Kingdom (McElroy et al., 2019, 2021). However, it is important to point out some limitations of the generalisability of our study. First, we excluded those who were unemployed during the entire observation period (2013–2018), as they could not respond to job-related questions. Secondly, as with any longitudinal study, the Swiss Household Panel suffers from attrition and non-response, both of which tend to occur to a greater extent among economically disadvantaged individuals (Rothenbühler & Voorpostel, 2016). We attempted to deal with this limitation by including individuals with at least one measure of each variable within the 6-year observation period and by imputing the missing information (Stekhoven & Buhlmann, 2012; Waljee et al., 2013). Nonetheless, it is still likely that, on average, participants of our study have higher income, better education, high-status jobs, and possibly, mental health and wellbeing than the general population in Switzerland. Importantly, however, age comparisons still can be considered valid due to similar patterns in missing information in both age groups.

## Conclusion

Middle-aged (age 40–55) individuals reported lower life satisfaction, joy, and higher anger, sadness, and worry than young adults (age 25–39). They also reported lower social support, relationships satisfaction, and health satisfaction – important correlates of mental health and wellbeing. Based on our findings, we propose self-perceived physical health, social relationships, including social support, and job demands as potential mechanisms explaining worse mental health and wellbeing in midlife. These factors are typically considered modifiable, hence may serve as targets for a public health interventions. Network analysis is a promising approach to studying complex processes, such as spillover effects in multiple life domains, which affect mental health and wellbeing. The network comparison across different ages can help us to speculate how these processes differ over time, which can be translated into hypotheses testable with confirmatory methodological approaches. For instance, our network model suggest that physical activity is interlinked with wellbeing and satisfaction with relationships in midlife, but not in young adulthood, hence potentially having spillover effects only in this age group.

### Supplementary Information

Below is the link to the electronic supplementary material.

Supplementary file1 (DOCX 2.23 MB)

## Data Availability

The data from the Swiss Household Panel is freely accessible to the scientific community on SWISSUbase.
