# Supplementary material for: Why do Middle-Aged Adults Report Worse Mental Health and Wellbeing than Younger Adults? An Exploratory Network Analysis of the Swiss Household Panel Data
Source: Appl Res Qual Life. 2024 Apr 4;19(4):1459–500. doi: 10.1007/s11482-024-10274-4 (PMC11349807; doi:10.1007/s11482-024-10274-4)
Supplement: Supplementary file 1 — Supplementary file1 (DOCX 2.23 MB) [file 11482_2024_10274_MOESM1_ESM.docx]

# Supplementary material

eText 1. Description of centrality stability and edge weight accuracy.

eTable 1. Predictors of having any missing information in the study sample.

eFigure 3. The network models, corrected for life stressors, for young and middle-aged adults.

eFigure 4. Differing pathways between young and middle-aged adults in network models corrected for life stressors.

eText 2. Description of a hypothetical explanatory study.

## eText 1. Description of centrality stability and edge weight accuracy.


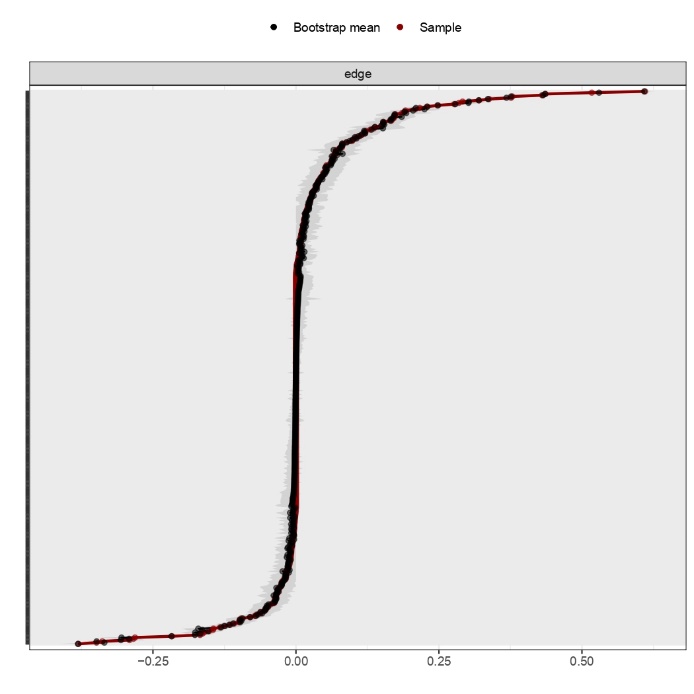

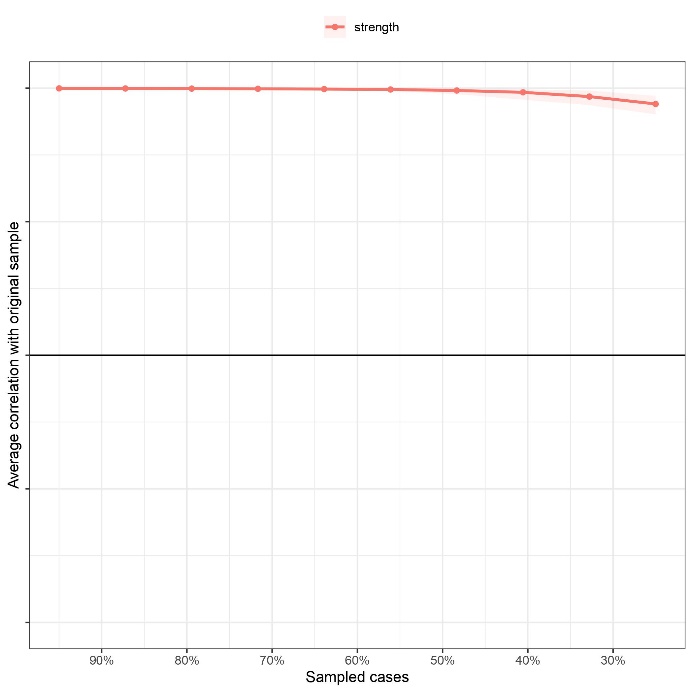


eFigure 2. Edge weight accuracy.

eFigure 1. Centrality stability.

Centrality stability refers to the reliability of the rank ordering of the centrality indices. This was assessed using the case-dropping subset bootstrap method (Epskamp et al., 2018); networks were re-estimated using increasingly smaller subsets of the original sample, and correlations between the original centrality indices and the subset centrality indices were calculated and plotted (eFigure 1). A small-to-moderate decrease in correlation as participants are removed indicates that the order of centrality is relatively stable or reliable. This can be quantified as the correlation stability coefficient, with values above 0.7 considered as high centrality reliability, and values between 0.25 and 0.7 deemed moderate reliability (Epskamp et al., 2018).

Edge weight accuracy refers to the degree of confidence with which we can interpret the ranking of the edge weights (strongest to weakest). To evaluate the accuracy of the networks, we calculated bootstrapped 95% confidence intervals for each edge, and plotted the results (eFigure 2). A lack of overlap between confidence intervals indicates a significant difference in the strength of two edges (Epskamp et al., 2018).

| eTable 1. Predictors of having any missing information in the study sample. | | |
| --- | --- | --- |
| Outcome: any missing information (vs not missing) | Risk ratio | CI 95% |
| Age |  |  |
| Young adulthood (25-39 years) | - | - |
| Middle adulthood (40-55 years) | 0.96 | (0.90, 1.01) |
| Gender |  |  |
| Women | - | - |
| Men | 1.05 | (0.99, 1.11) |
| Nationality |  |  |
| Swiss | - | - |
| Non-Swiss | 2.11 | (2.01, 2.22) |
| Language of interview |  |  |
| French | - | - |
| German | 0.91 | (0.83, 1.00) |
| Italian | 1.15 | (0.97, 1.36) |
| Life satisfaction | 0.98 | (0.96, 1.00) |
| Years of education | 0.96 | (0.94, 0.97) |
| Household income (log-transformed) | 0.68 | (0.68, 0.68) |
| Social support | 0.97 | (0.95, 1.00) |
| Trust in people | 0.95 | (0.94, 0.96) |


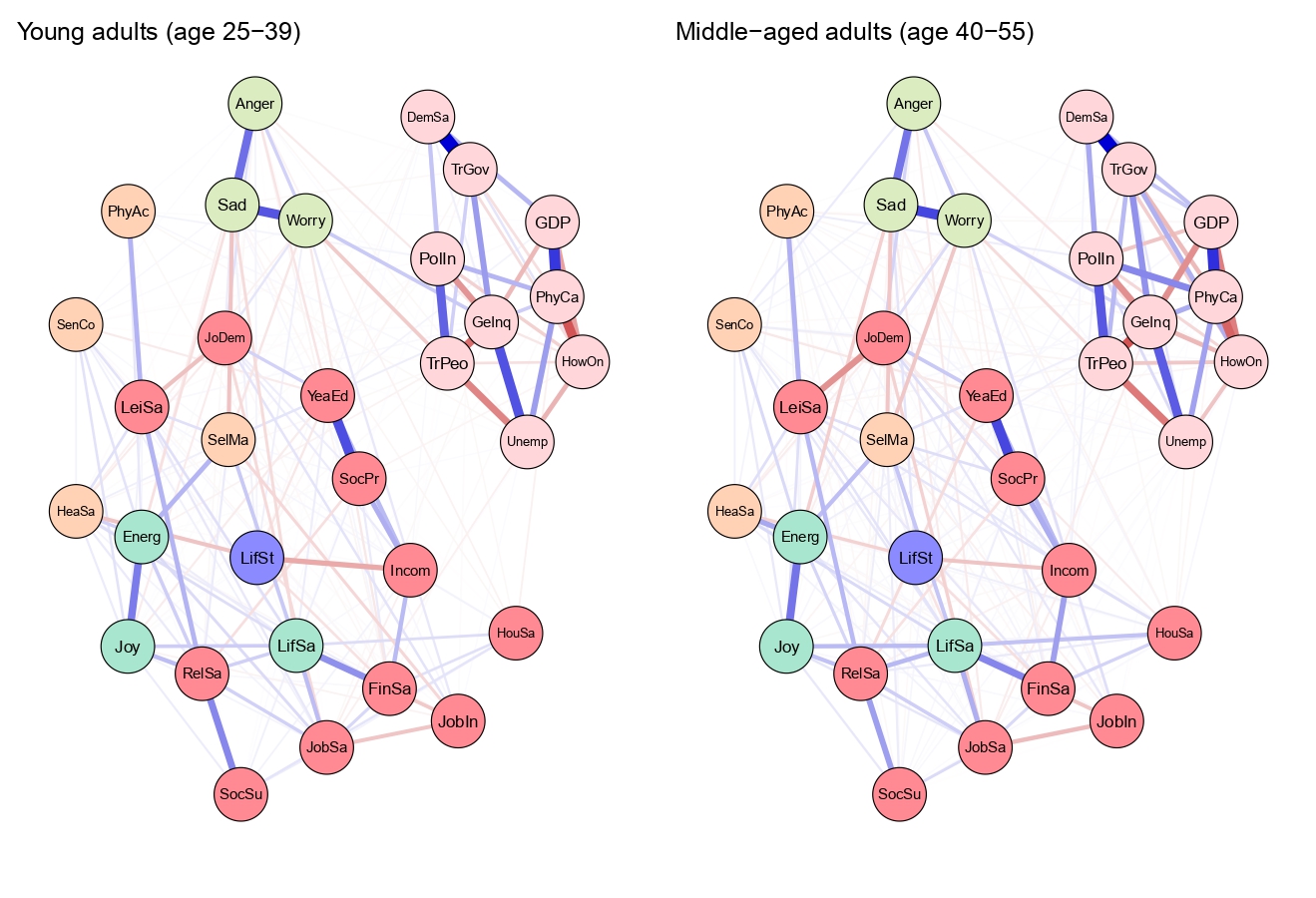


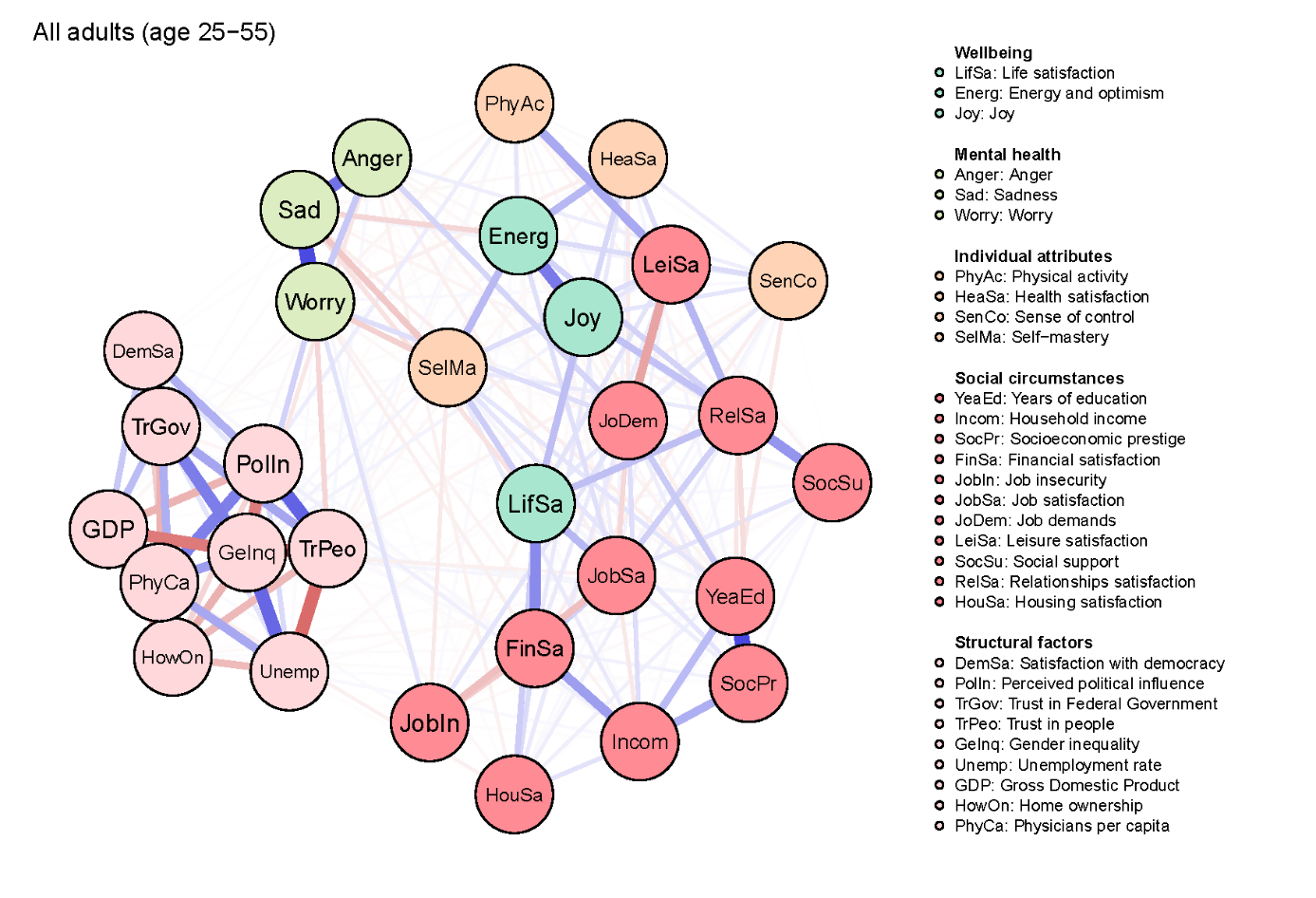


eFigure 3. The network models, corrected for life stressors, for young and middle-aged adults.

Legend: blue lines = positive interrelations; red lines = negative interrelations.


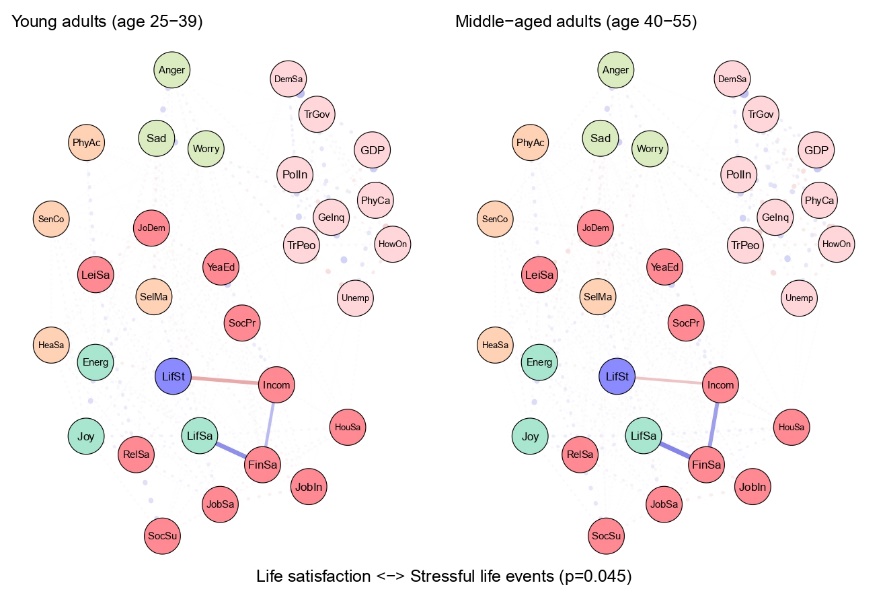

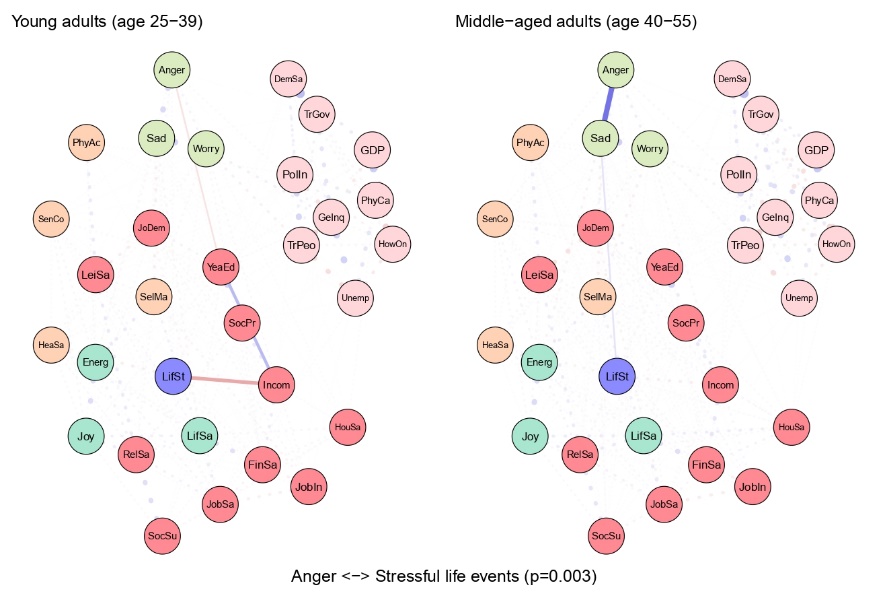

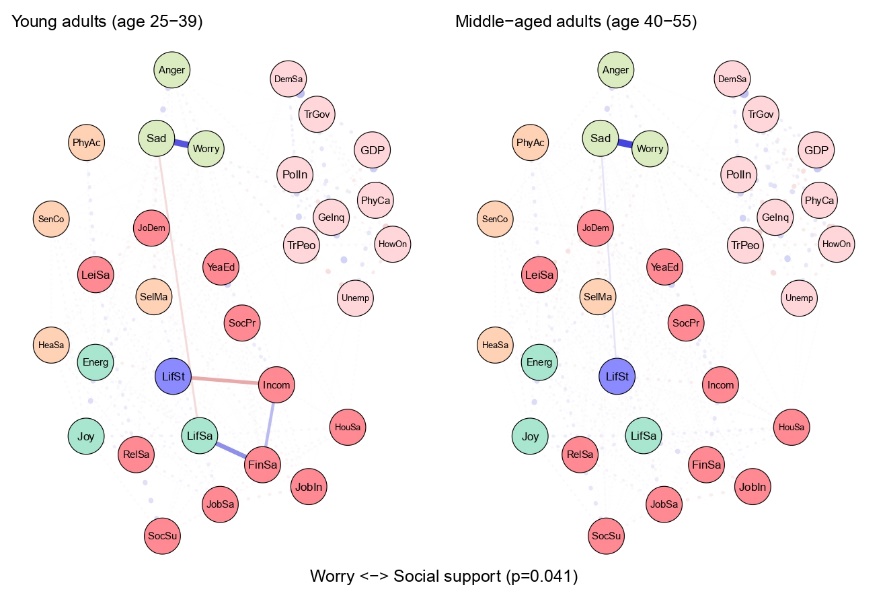


eFigure 4. Differing pathways between young and middle-aged adults in network models corrected for life stressors.

Legend: non-transparent, continuous blue lines = positive interrelations; non-transparent, continuous red lines = negative interrelations; transparent, dotted lines = all remaining partial regularized correlation relationships.

## eText 2. Description of a hypothetical explanatory study.


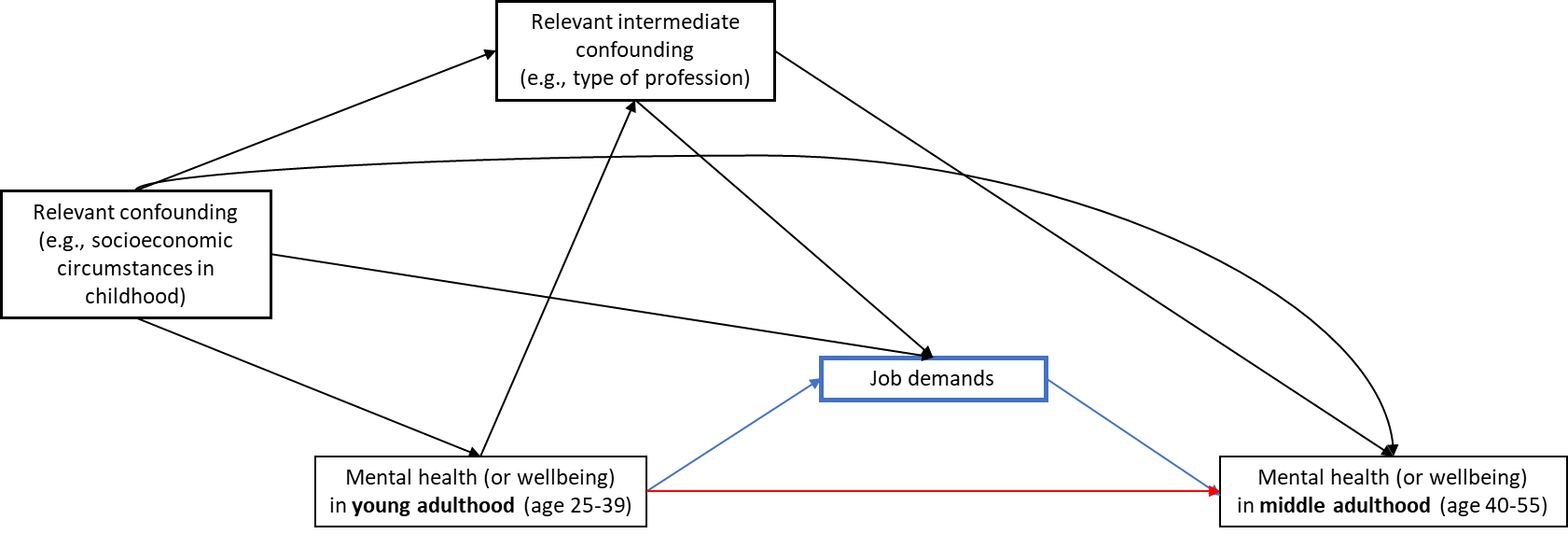


eFigure 5. The directed acyclic graph (DAG) of a potential causal mediation analysis examining mechanisms that may explain poorer mental health or wellbeing in midlife than in young adulthood.

This directed acyclic graph (DAG) presents a conceptual representation of a causal mediation analysis that would examine the extent to which physical health, social relationships (and support) and job demands could explain why mental health and wellbeing deteriorate between young and middle adulthood. Here, we take job demands as an example, but a similar analysis could be conducted with social relationships (and support) and job demands. A hypothetical study could examine all three mechanisms simultaneously under additional assumptions, that is, taking into consideration the causal structure between the mediators (e.g., whether job demands cause poor social relationships or vice versa).

We propose that future research uses longitudinal studies, with a sufficiently long follow-up to cover the period between young and middle adulthood (age 25-55), to examine intraindividual change in mental health or wellbeing. Ideally, such a study would include more than one birth cohort to be able to examine potential cohort effects on interindividual change. For instance, the 1946 and 1958 British birth cohorts could be combined (Power & Elliott, 2006; Wadsworth et al., 2005). In such a study, the direct and indirect effects of mental health (or wellbeing) in young adulthood on mental health (or wellbeing) in midlife would be estimated, that is, they would capture the change in mental health (or wellbeing) between these age periods). The indirect effect (blue arrows) would be defined as the effect of mental health (or wellbeing) in young adulthood mediated via the proposed mechanisms, job demands in this case, on mental health (or wellbeing) in midlife. Then the mediated proportion of the total effect could be estimated, which is the proportion of indirect effect out of summed indirect and direct (red line) effects. This would help us to judge the potential benefits of intervening on the explanatory factors, job demands, in this case. The mediation causal analysis would need to meet the relevant assumptions (more details can be found elsewhere (Rijnhart et al., 2021), for instance, accounting for confounding as depicted in the figure.

Epskamp, S., Borsboom, D., & Fried, E. I. (2018). Estimating psychological networks and their accu racy: a tutorial paper. *Behav Res Methods.*, *50*, 195-212.

Power, C., & Elliott, J. (2006). Cohort profile: 1958 British birth cohort (National Child Development Study). *Int J Epidemiol*, *35*(1), 34-41. <https://doi.org/10.1093/ije/dyi183>

Rijnhart, J. J. M., Lamp, S. J., Valente, M. J., MacKinnon, D. P., Twisk, J. W. R., & Heymans, M. W. (2021). Mediation analysis methods used in observational research: a scoping review and recommendations. *BMC Medical Research Methodology*, *21*(1), 226. <https://doi.org/10.1186/s12874-021-01426-3>

Wadsworth, M., Kuh, D., Richards, M., & Hardy, R. (2005). Cohort Profile: The 1946 National Birth Cohort (MRC National Survey of Health and Development). *International Journal of Epidemiology*, *35*(1), 49-54. <https://doi.org/10.1093/ije/dyi201>
